# Supplementary material for: Not just words! Effects of a light-touch randomized encouragement intervention on students’ exam grades, self-efficacy, motivation, and test anxiety
Source: PLoS One. 2021 Sep 15;16(9):e0256960. doi: 10.1371/journal.pone.0256960 (PMC8443032; doi:10.1371/journal.pone.0256960)
Supplement: S1 Appendix — (DOCX) [file pone.0256960.s001.docx]

**S1 Appendix: Students’ perceptions of the intervention**

The appendix belongs to the following paper by **Tamás Keller** and **Péter Szakál**:

Not just words! Effects of a light-touch randomized encouragement intervention on students’ exam grades, self-efficacy, motivation, and test anxiety

Five months after our encouragement campaign (in May 2020), we invited all the students in our analytic sample to participate in an online survey about their experiences with our campaign. Approximately 16% of our population answered the survey (N = 2,420).

Ideally, the online follow-up survey should be administered earlier, immediately after the treatment. It was not feasible, however, due to the closures and switch to online education caused by the COVID-19 pandemic. These changes challenged the university’s online platform and required the full attention of the administrative staff who could administer the infrastructure of such an online survey.

Although five months is a significant amount of time and students’ memories might be attenuated, 79% of the respondents correctly recalled the content of the message, while 9.5% of students claimed not to remember. The rest of the respondents either did not answer the question (6.5%) or recalled incorrect content (5%). These figures indicate that students’ memories about the intervention had not attenuated significantly by the time of the follow-up survey.

The retrospective online survey provides us with qualitative information; in particular on the reception of the campaign, on possible adverse effects, and on the magnitude of treatment contamination.

First, students on average positively evaluated the encouragement campaign. On a five-point Likert scale, 65% of students answered that they were “happy” or “very happy” when they received the encouragement, and 77% of students stated that they would like to receive similar encouragement messages in the future.

Second, students who had not received the encouragement message might have been discouraged. On a five-point Likert-scale, 17% of students indicated that they were “sad” or “very sad” when we had asked them the question: “How sad were you when you found out that your peers had received the encouragement message, but you had not?” The discouragement of untreated students may lead to adverse treatment effects. It moves our estimations into the anticonservative direction, as while the treatment boosts the outcomes of treated students, the absence of treatment can worsen control students’ outcomes. However, our qualitative data suggest that the magnitude of the adverse treatment effect might be moderate.

Third, approximately one-third of students may have been informed about the treatment before receiving it. In particular, 37% of students had heard that fellow students had received encouragement messages at a time when the students themselves had not yet received it, and 33% of students shared the message with their peers at the university after they had received it. Consequently, our treatment may have lost its novelty over time. Therefore, students treated later might have experienced a smaller treatment effect. Nevertheless, this makes our estimation more conservative.

It is important to note that these two facts (first that some students were informed about the treatment before being treated, and second that some students shared the treatment with their peers) should not be interpreted as indications of spillover effects. It is rather a form of treatment contamination. Our treatment is if someone actually received the encouragement message and not when students have just heard about the existence of the treatment. In this regard, prior information about the existence of the encouragement messages is less relevant.
