# Supplementary material for: Not just words! Effects of a light-touch randomized encouragement intervention on students’ exam grades, self-efficacy, motivation, and test anxiety
Source: PLoS One. 2021 Sep 15;16(9):e0256960. doi: 10.1371/journal.pone.0256960 (PMC8443032; doi:10.1371/journal.pone.0256960)
Supplement: S2 Appendix — (DOCX) [file pone.0256960.s002.docx]

**S2 Appendix: Subsamples**

The appendix belongs to the following paper by **Tamás Keller** and **Péter Szakál**:

Not just words! Effects of a light-touch randomized encouragement intervention on students’ exam grades, self-efficacy, motivation, and test anxiety

We gathered data about students with two questionnaires. Students voluntarily answered these questionnaires; therefore, the variables collected via the survey do not have full coverage but are only available for a subsample of students. Column 1 in the table below shows the composition of the full target population regarding students’ baseline characteristics.

As Column 2 in the table shows, almost half of the full sample (45%) filled in the endline survey (N = 7,026). Compared to those who did not fill in the endline survey (Column 4 in the table below), the subsample of students that filled in the survey is more advantaged. It contains younger and more able students who are more likely to be enrolled in full-time and state-financed education, and the proportion of female students is also over-represented among them. Most importantly, however, the share of students who were allocated to Group A is the same in the two groups. Despite these differences, the treatment effect on students’ endline exam grades among those who answered the endline questionnaire is qualitatively similar to the results shown in the paper (Table A4 in S7 Appendix).

Those students who filled in the endline questionnaire twice (Column 5 in the table below) make up a younger and more able subsample of students than those who filled in the endline survey once (Column 7 in the table below). Those allocated to Group A have a 3 percentage-point higher likelihood of answering the endline survey twice. This difference is statistically significant at the 5% significance level.

The treatment status significantly decreased students’ willingness to answer the endline questionnaire, both before students’ first and second exams by 3.6 and 5.2 percentage points, respectively. As the e-mail that the control students received prompted them to go directly to the lottery, control students received stronger incentives to participate in the survey and win, which might explain why control students were more likely to fill in the endline survey. This type of selection could undermine the results on the secondary outcomes. Nevertheless, as Tables A6 to A8 the in S7 Appendix show, the estimations were qualitatively similar among those who answered the endline questionnaire twice, and thus filled in the questionnaire in the treated and also in the control condition.

A small subsample of students (15%) answered the baseline background questionnaire (Column 8 in the table below). The subsample of these students is more advantaged than those who did not fill in the baseline background questionnaire (Column 10 in the table below). However, there is no difference between the two groups in terms of the allocation of students to Group A.

In sum, the composition of subsamples differs in terms of the baseline covariates. Across different subsamples, however, the share of students allocated to different treatment groups is similar, except for those who filled in the endline survey twice versus once. In this case, however, the difference is quite small (3 percentage points), and it is below the preregistered threshold.

**Differences between the composition of various sub-samples**

|  | (1) | (2) | (3) | | (4) | | (5) | | (6) | (7) | | (8) | | (9) | (10) | |
| --- | --- | --- | --- | --- | --- | --- | --- | --- | --- | --- | --- | --- | --- | --- | --- | --- |
|  | All students | Endline Questionnaire | | | | | Has filled in Endline Questionnaire | | | | | Baseline Questionnaire on psychological variables  (see S4 Appendix) | | | | |
|  |  | Filled in the | | Did not fill in | | Diff. | Twice | Once | | | Diff | Filled in | Did not fill in | | | Diff |
| Group A | 0.50 | 0.50 | | 0.50 | | 0.00 | 0.53 | 0.50 | | | 0.03* | 0.50 | 0.50 | | | 0.00 |
| Has two exams | 0.92 | 0.94 | | 0.90 | | 0.05* | 1.00 | 0.93 | | | 0.07* | 0.95 | 0.91 | | | 0.04* |
| Has two endline survey data | 0.09 | 0.20 | | n.a. | | n.a. | 1.00 | 0.00 | | |  | 0.19 | 0.08 | | | 0.11* |
| Female | 0.57 | 0.59 | | 0.56 | | 0.03* | 0.60 | 0.59 | | | 0.01 | 0.64 | 0.56 | | | 0.07* |
| Age | 24.12 | 23.94 | | 24.26 | | -0.32* | 24.06 | 23.91 | | | 0.15 | 23.64 | 24.20 | | | -0.56* |
| Students’ ability^a^ | 0.00 | 0.03 | | -0.02 | | 0.05* | 0.07 | 0.02 | | | 0.06* | 0.02 | 0.00 | | | 0.02 |
| Students’ ability is missing | 0.32 | 0.30 | | 0.33 | | -0.02* | 0.28 | 0.31 | | | -0.03* | 0.29 | 0.32 | | | -0.03* |
| Full-time training | 0.83 | 0.84 | | 0.81 | | 0.02* | 0.84 | 0.84 | | | -0.01 | 0.87 | 0.82 | | | 0.05* |
| State-financed training | 0.75 | 0.77 | | 0.73 | | 0.04* | 0.78 | 0.77 | | | 0.01 | 0.81 | 0.74 | | | 0.07* |
| Bachelor level | 0.59 | 0.59 | | 0.59 | | 0.01 | 0.62 | 0.59 | | | 0.03* | 0.56 | 0.59 | | | -0.03* |
| Master level | 0.09 | 0.09 | | 0.10 | | -0.01* | 0.08 | 0.09 | | | -0.01 | 0.09 | 0.09 | | | 0.00 |
| Undivided | 0.24 | 0.25 | | 0.24 | | 0.01 | 0.23 | 0.25 | | | -0.03* | 0.29 | 0.24 | | | 0.06* |
| Higher-level vocational training | 0.08 | 0.07 | | 0.08 | | -0.01 | 0.08 | 0.07 | | | 0.01 | 0.05 | 0.08 | | | -0.03* |
| First-year student | 0.31 | 0.33 | | 0.30 | | 0.03* | 0.36 | 0.32 | | | 0.04* | 0.33 | 0.30 | | | 0.03* |
| Exam difficulty | 0.19 | 0.20 | | 0.19 | | 0.01* | 0.22 | 0.19 | | | 0.02* | 0.18 | 0.19 | | | -0.01* |
| Exam difficulty is missing | 0.19 | 0.17 | | 0.20 | | -0.03* | 0.16 | 0.17 | | | -0.01 | 0.17 | 0.19 | | | -0.02* |
| Baseline test anxiety^a^ |  |  | |  | |  |  |  | | |  | 0.00 |  | | |  |
| Baseline self-confidence^a^ |  |  | |  | |  |  |  | | |  | 0.00 |  | | |  |
| Baseline external control^a^ |  |  | |  | |  |  |  | | |  | 0.00 |  | | |  |
| Parental education (university degree |  |  | |  | |  |  |  | | |  | 0.54 |  | | |  |
| N | 15,539 | 7,026 | | 8,513 | |  | 1,433 | 5,593 | | |  | 2,305 | 13,234 | | |  |
| % | 100.00% | 45.22% | | 54.78% | |  | 9.22% | 35.99% | | |  | 14.83% | 85.17% | | |  |

* The difference is significant at 5% level using a two-tailed t-test.

^a^ z-standardized variable at 0 mean and 1 standard deviation
