# Supplementary material for: Not just words! Effects of a light-touch randomized encouragement intervention on students’ exam grades, self-efficacy, motivation, and test anxiety
Source: PLoS One. 2021 Sep 15;16(9):e0256960. doi: 10.1371/journal.pone.0256960 (PMC8443032; doi:10.1371/journal.pone.0256960)
Supplement: S3 Appendix — (DOCX) [file pone.0256960.s003.docx]

**S3 Appendix: Descriptive statistics of the outcome variables in the whole sample and in the subsample of those who answered the endline questionnaire**

The appendix belongs to the following paper by **Tamás Keller** and **Péter Szakál**:

Not just words! Effects of a light-touch randomized encouragement intervention on students’ exam grades, self-efficacy, motivation, and test anxiety

The 15,539 students in the analytical sample have a total of 28,156 exam grades, out of which 14,673 grades are from the first exams, and 13,483 grades from the second exams. There are 866 students (5.57%) who did not take the first exam (for whatever reason, most likely because they became ill). 2,056 students (13.23%) did not take a second exam since they had only one exam, or because they became ill before the second exam. The allocation of students to Group A has no effect on missing endline grades in the first (coef. = -0.004; p = 0.232) and second exams (coef. = -0.009; p = 0.09).

There are 7,026 students who filled in the endline survey; 20.4% of them answered the endline questionnaire twice before their first and second exams as well. Thus, there are 8,459 valid answers to the endline questionnaire. The majority of answers were filled in before students’ first exams (64%, N = 5,414) and about one third were filled in before students’ second exams (36%, N = 3,045). The allocation of students to Group A has no effect on students’ participation in the survey (coef. = -0.002; p = 0.764).

**Descriptive statistics of the outcome variables in the whole sample, and in the subsample of those who answered the endline questionnaire**

|  | **Exam grades** | **Exam grades** | **Test anxiety** | **Self-efficacy** | **Motivation** |
| --- | --- | --- | --- | --- | --- |
| Panel A: Both exams | *Whole sample*  *N=31,078* | *The subsample of those who answered the endline questionnaire*  *N=8,459, from 7,026 students* | | | |
| Mean | 3.559 | 3.545 | 6.367 | 5.899 | 9.011 |
| SD | 1.454 | 1.438 | 2.913 | 2.526 | 1.532 |
| N of non-missing | 28,156 | 8,158 | 8,316 | 8,296 | 8,301 |
| N of missing | 2,922 | 301 | 143 | 163 | 158 |
| Panel B: First exam | *Whole sample*  *N=15,539* | *The subsample of those who answered the endline questionnaire*  *N=5,414* | | | |
| Mean | 3.636 | 3.648 | 6.373 | 6.000 | 9.032 |
| SD | 1.425 | 1.405 | 2.889 | 2.477 | 1.494 |
| N of non-missing | 14,673 | 5,226 | 5,325 | 5,310 | 5,318 |
| N of missing | 866 | 188 | 89 | 104 | 96 |
| Panel C: Second exam | *Whole sample*  *N=15,539* | *The subsample of those who answered the endline questionnaire*  *N=3,045* | | | |
| Mean | 3.476 | 3.362 | 6.356 | 5.719 | 8.973 |
| SD | 1.480 | 1.478 | 2.957 | 2.602 | 1.595 |
| N of non-missing | 13,483 | 2,932 | 2,991 | 2,986 | 2,983 |
| N of missing | 2,056 | 113 | 54 | 59 | 62 |
