# Supplementary material for: Not just words! Effects of a light-touch randomized encouragement intervention on students’ exam grades, self-efficacy, motivation, and test anxiety
Source: PLoS One. 2021 Sep 15;16(9):e0256960. doi: 10.1371/journal.pone.0256960 (PMC8443032; doi:10.1371/journal.pone.0256960)
Supplement: S4 Appendix — (DOCX) [file pone.0256960.s004.docx]

**S4 Appendix: Control variables**

The appendix belongs to the following paper by **Tamás Keller** and **Péter Szakál**:

Not just words! Effects of a light-touch randomized encouragement intervention on students’ exam grades, self-efficacy, motivation, and test anxiety

We preregistered to deploy the following baseline variables to increase the precision of the estimation. The source of the baseline variables was the university’s administrative registry information about students.

1. Students’ *gender* can be either male or female. Females are coded as 1.
2. Students’ *age* is the difference between the date of the exam and their date of birth divided by 365.
3. Students’ *ability* is measured by students’ high-school final examination test scores, which also serves as the admission score to tertiary education. In the high-school final examination, all students sit the same standardized tests (exams) in their chosen subjects. There are subjects like mathematics or Hungarian, for which taking the high-school final examination is mandatory. However, some study programs at the university might require students to take the high-school final examination in particular subjects. For example, not all students have to take high school final examinations in biology, but students applying to become medical doctors have to take this exam. Our measure relates to the students’ tests scores in the subjects that a particular study program expected them to take. Thus, our measure of ability is specific to the particular study program and field of study. The ability variable is z-standardized at 0 mean and 1 standard deviation.

We assume that students’ admission score is a better proxy for students’ abilities than their last semester GPA. Students’ GPA in prior semesters is the outcome of the university’s grading standard. It is hard to compare, therefore, students’ GPAs between different faculties and disciplines. By contrast, the test scores of high school final examinations are nationally standardized tests and thus serve as a universal standard of comparison.

Furthermore, students’ GPA incorporates their motivations and effort. Thus, their prior GPA is also a response by students to those endogenous shocks that they experienced at the university (like how much students like their subjects/university). Student admission scores are, however, by definition, not an effect of those endogenous shocks experienced at the university. Results are robust, however, for controlling for students’ prior GPA instead of their admission scores (Table A9 in the S7 Appendix).

1. *Type of training* is a dummy variable indicating whether a student is engaged in full-time training (=1) or either correspondence training or distance learning (=0).
2. The *financial form of training* is coded as state-financed (=1) or self-financed (=0).
3. The *level of training* is coded as bachelor level (=1), master level (=2), undivided (=3), and higher-level vocational training (4). Undivided programs are the kind of program commonly offered for medical doctors or lawyers, where there is no division between BA and upper levels of the program. Higher-level vocational training involves two years of training taught in higher educational institutions and results in a qualification similar to a bachelor's degree, although the qualification itself is not a degree.
4. A dummy variable *first-year student* indicates (=1) whether students are freshmen or more long-standing students at the university (=0).
5. The *difficulty of the exam* is defined as the proportion of those who failed the respective exam in the last semester.
6. We employ *study program* (for example, sociology) fixed effects. Study programs are assigned to university faculties such as the Faculty of Social Science.

We preregistered to replace missing values in control variables with 0. Separate dummy variables control for the missing status data in each variable so as not to lose observations.

In addition to the registry data, we sent out a baseline background questionnaire to all students in the population. We inquired into psychological variables and parental background information in the questionnaire. In particular, we deployed the following questions in the baseline questionnaire:

1. We asked students about their *baseline test anxiety* by using items from the Sarason [1] test anxiety scale. This is a z-standardized variable at 0 mean and 1 standard deviation.
2. We collected data about *baseline self-confidence* by applying the academic subscale items from Shrauger and Schohn’s [2] Personal Evaluation Inventory. This is a z-standardized variable at 0 mean and 1 standard deviation.
3. Locus of control measures the sense of agency people feel over their lives. Locus of control is believed to be conceptually similar to self-efficacy [3] and is conceptually connected to behavioral intention and control in Ajzen’s theory of planned behavior [4]. We measured the baseline external/internal *locus of control* [5] using the four-item version of the Rotter-scale test [6,7]. In the test, respondents choose between two sentences describing external and internal control conditions. People with an internal locus of control believe that their abilities and actions influence their life outcomes. By contrast, people with an external locus of control believe that random chance and environmental factors affect their life outcomes. Throughout the analysis, we used a scale of external control in which we calculated the sum of those answers in which respondents opted for the external control options. We have a z-standardized variable at 0 mean and 1 standard deviation.
4. We asked about parental education by deploying separate seven-grade questions for the mother’s and father’s highest educational level. Parental education is coded as 1 if either the father or mother has a university degree.

The corresponding survey instruments are shown in the original Hungarian in S8 Appendix and in Englis in S9 Appendix.

Further decisions for coding of the baseline variables are specified in the pre-analysis plan.

Some of the control variables served the purpose of testing treatment heterogeneity. The variables listed below are those baseline variables that served the purpose of testing treatment heterogeneity but were not deployed as control variables.

*First-year student*: is a dummy variable indicating whether the student is a first-year student (=1). These students had no prior exam experience at the university; thus, their first exam has particular importance. The variable is coded 0 if the student is not a first-year student.

*Has mobile phone*: is a dummy variable indicating whether a student had a mobile phone number stored in the university’s register (=1). The variable is coded 0 when it indicates the absence of a mobile phone number in the university’s register.

*Day of message*: This is a number ranging from 1 (the first day of the campaign) until the last day when students are treated.

**References**

1. Sarason IG. Test Anxiety: Theory, Research, and Applications. Hillsdale, NJ: Lawrence Erlbaum Associates; 1980. 193–216 p. Available from: http://www.mrc.stlmath.com/pdf/anxiety/scale.pdf

2. Shrauger JS, Schohn M. Self-Confidence in College Students: Conceptualization, Measurement, and Behavioral Implications. Assessment. 1995 Sep 26;2(3):255–78. Available from: http://journals.sagepub.com/doi/10.1177/1073191195002003006

3. Rotter JB. Some comments on the “Cognates of personal control.” Appl Prev Psychol. 1992;1(2):127–9.

4. Ajzen I. Perceived Behavioral Control, Self-Efficacy, Locus of Control, and the Theory of Planned Behavior. J Appl Soc Psychol. 2002 Apr;32(4):665–83. Available from: http://doi.wiley.com/10.1111/j.1559-1816.2002.tb00236.x

5. Rotter JB. Generalized expectancies for internal versus external control of reinforcement. Psychol Monogr Gen Appl. 1966;80(1):1–28. Available from: http://doi.apa.org/getdoi.cfm?doi=10.1037/h0092976

6. Andrisani PJ. Internal-External Attitudes, Personal Initiative, and the Labor Market Experience of Black and White Men. J Hum Resour. 1977;12(3):308. Available from: https://www.jstor.org/stable/145493?origin=crossref

7. Goldsmith AH, Veum JR, William D. The impact of labor force history on self-esteem and its component parts, anxiety, alienation and depression. J Econ Psychol https://linkinghub.elsevier.com/retrieve/pii/0167487096000037
