# Supplementary material for: Not just words! Effects of a light-touch randomized encouragement intervention on students’ exam grades, self-efficacy, motivation, and test anxiety
Source: PLoS One. 2021 Sep 15;16(9):e0256960. doi: 10.1371/journal.pone.0256960 (PMC8443032; doi:10.1371/journal.pone.0256960)
Supplement: S5 Appendix — (DOCX) [file pone.0256960.s005.docx]

**S5 Appendix: Pairwise correlation between various psychological measures and the secondary outcome variables**

The appendix belongs to the following paper by **Tamás Keller** and **Péter Szakál**:

Not just words! Effects of a light-touch randomized encouragement intervention on students’ exam grades, self-efficacy, motivation, and test anxiety

In order to ensure a high response rate in the endline questionnaire, we only deployed a few questions. Since students were asked to respond to the survey in a stressful situation—before their exam—we could not deploy detailed measures containing many items in the questionnaire.

We tested how our simple single-item endline variables correlated with the more detailed baseline variables. We found a pairwise correlation of 0.48 (p<0.01) between students’ endline test anxiety measured by the single-item question, and the more detailed measure for baseline test anxiety using items from the Sarason’ test anxiety scale [1].

Similarly, we found a pairwise correlation coefficient of 0.42 (p<0.01) between the single-item endline measure of endline self-efficacy and the more detailed measure of baseline self-confidence, deploying the academic subscale items from Shrauger and Schohn’s Personal Evaluation Inventory [2].

Thus, we concluded that our endline questions are good proxies of the underlying ability beliefs.

**Pairwise correlation between various psychological measures and the secondary outcome variables**

|  | Baseline test anxiety | Endline test anxiety | Baseline self-confidence | Endline self-efficacy | Endline motivation |
| --- | --- | --- | --- | --- | --- |
| Endline test-anxiety | 0.4769* |  |  |  |  |
|  | (1,042) |  |  |  |  |
| Baseline self-confidence | -0.4906* | -0.2685* |  |  |  |
|  | (2,305) | (1,042) |  |  |  |
| Endline self-efficacy | -0.3505* | -0.2248* | 0.4178* |  |  |
|  | (1,044) | (4,284) | (1,044) |  |  |
| Endline motivation | 0.0148 | 0.2782* | 0.0794 | 0.2729* |  |
|  | (1,042) | (4,283) | (1,042) | (4,289) |  |
| External locus of control | 0.1803* | 0.0781 | -0.1796* | -0.1542* | -0.0584 |
|  | (2,305) | (1,042) | (2,305) | (1,044) | (1,042) |

Pairwise correlation coefficients, N of cases in parenthesis, * p<0.01

***Baseline test anxiety*** is measured by using items from the Sarason’ test anxiety scale.

***Baseline self-confidence*** is measured by using the academic subscale items from Shrauger and Schohn’s Personal Evaluation Inventory.

***External locus of control*** is measured by the four-item version of the test Rotter scale.

Endline measures are measured by single-item questions.

**References**

1. Sarason, Irwin G. 1980. *Test Anxiety: Theory, Research, and Applications*. Hillsdale, NJ: Lawrence Erlbaum Associates. http://www.mrc.stlmath.com/pdf/anxiety/scale.pdf.
2. Shrauger, J. Sidney, and Mary Schohn. 1995. “Self-Confidence in College Students: Conceptualization, Measurement, and Behavioral Implications.” *Assessment* 2(3): 255–78. http://journals.sagepub.com/doi/10.1177/1073191195002003006.
