# Supplementary material for: Not just words! Effects of a light-touch randomized encouragement intervention on students’ exam grades, self-efficacy, motivation, and test anxiety
Source: PLoS One. 2021 Sep 15;16(9):e0256960. doi: 10.1371/journal.pone.0256960 (PMC8443032; doi:10.1371/journal.pone.0256960)
Supplement: S6 Appendix — (DOCX) [file pone.0256960.s006.docx]

**S6 Appendix: Results of the alternative model specifications**

The appendix belongs to the following paper by **Tamás Keller** and **Péter Szakál**:

Not just words! Effects of a light-touch randomized encouragement intervention on students’ exam grades, self-efficacy, motivation, and test anxiety

**List of tables in the document**

[**Table A1: Robustness check: main treatment effect with exam-subject fixed effects on the four endline outcome variables in the case of the first exam** 2](#_Toc81401451)

[**Table A2: Robustness check: main treatment effect with exam-subject fixed effects on the four endline outcome variables in the case of the second exam** 3](#_Toc81401452)

[**Table A3: Robustness check: main treatment effect with student fixed effects on the four endline outcome variables** 4](#_Toc81401453)

We could not include exam-subject fixed effects in the preregistered equation (Eq.1) since this would be collinear with the preregistered exam dummy (E) indicating students’ first and second exam.

As a robustness check, we show alternative models using exam-subject fixed effects and restricting the sample to students’ first (Table A1) and second exams (Table A2).

Furthermore, we deploy students’ fixed effects to capture all unobserved differences at the student level (Table A3).

Our results are qualitatively the same as the results calculated with the preregistered model.

# **Table A1: Robustness check: main treatment effect with exam-subject fixed effects on the four endline outcome variables in the case of the first exam**

|  | Exam grades | Test anxiety | Self-efficacy | Motivation to do well on the exam |
| --- | --- | --- | --- | --- |
| Treated | 0.003 | -0.062 | 0.254** | 0.088+ |
|  | (0.018) | (0.092) | (0.078) | (0.049) |
| Constant | 3.866*** | 11.103* | 3.578 | 9.802** |
|  | (0.911) | (5.656) | (4.785) | (2.996) |
| N of students | 14,673 | 5,325 | 5,310 | 5,318 |
| Cohen’s *d* effect size | 0.00 | -0.02 | 0.10 | 0.06 |

All models contain the following preregistered standard baseline control variables: student’s gender, age, ability, student is a first-year student, the type of training, the financial form of training, the level of training, the difficulty of the exam, and study program fixed effects.

Standard errors in parentheses, *** p<0.001, ** p<0.01, * p<0.05, + p<0.1

# **Table A2: Robustness check: main treatment effect with exam-subject fixed effects on the four endline outcome variables in the case of the second exam**

|  | Exam grades | Test anxiety | Self-efficacy | Motivation to do well on the exam |
| --- | --- | --- | --- | --- |
| Treated | -0.014 | 0.122 | 0.245* | 0.005 |
|  | (0.020) | (0.136) | (0.116) | (0.077) |
| Constant | 1.687 | 10.541*** | 11.373*** | 11.348*** |
|  | (1.711) | (2.632) | (2.267) | (1.537) |
| N of students | 13,483 | 2,991 | 2,986 | 2,983 |
| Cohen’s *d* effect size | -0.01 | 0.04 | 0.09 | 0.00 |

All models contain the following preregistered standard baseline control variables: student’s gender, age, ability, student is a first-year student, the type of training, the financial form of training, the level of training, the difficulty of the exam, and study program fixed effects.

Standard errors in parentheses, *** p<0.001, ** p<0.01, * p<0.05, + p<0.1

# **Table A3: Robustness check: main treatment effect with student fixed effects on the four endline outcome variables**

|  | Exam grades | Test anxiety | Self-efficacy | Motivation to do well on the exam |
| --- | --- | --- | --- | --- |
| Treated | -0.010 | 0.001 | 0.227*** | 0.010 |
|  | (0.014) | (0.078) | (0.068) | (0.044) |
| Exam (second =1) | -0.010 | -0.113 | -0.030 | -0.074 |
|  | (0.019) | (0.113) | (0.098) | (0.063) |
| Constant | 104.030*** | -112.185 | 151.131* | -19.269 |
|  | (13.934) | (74.519) | (65.040) | (41.851) |
| N of students | 28,156 | 8,316 | 8,296 | 8,301 |
| Cohen’s *d* effect size | -0.01 | 0.00 | 0.09 | 0.01 |

All models contain the following preregistered standard baseline control variables: student’s gender, age, ability, student is a first-year student, the type of training, the financial form of training, the level of training, the difficulty of the exam, and study program fixed effects.

Standard errors in parentheses, *** p<0.001, ** p<0.01, * p<0.05, + p<0.1
