# Supplementary material for: Not just words! Effects of a light-touch randomized encouragement intervention on students’ exam grades, self-efficacy, motivation, and test anxiety
Source: PLoS One. 2021 Sep 15;16(9):e0256960. doi: 10.1371/journal.pone.0256960 (PMC8443032; doi:10.1371/journal.pone.0256960)
Supplement: S7 Appendix — (DOCX) [file pone.0256960.s007.docx]

**S7 Appendix: Results of sensitivity analyses**

The appendix belongs to the following paper by **Tamás Keller** and **Péter Szakál**:

Not just words! Effects of a light-touch randomized encouragement intervention on students’ exam grades, self-efficacy, motivation, and test anxiety

**List of tables in the document**

[**Table A4.: Treatment effect on students’ endline exam grades, among those who answered the endline questionnaire 2**](#_Toc81401694)

[**Table A5.: Treatment effect on students’ endline exam grades, among students who have two endline exam-grades 4**](#_Toc81401695)

[**Table A6.: Treatment effect on students’ endline test anxiety among students who answered the endline survey twice 6**](#_Toc81401696)

[**Table A7.: Treatment effect on students’ endline self-efficacy among students who answered the endline survey twice 8**](#_Toc81401697)

[**Table A8.: Treatment effect on students’ endline motivation to do well on the exam among students who answered the endline survey twice 10**](#_Toc81401698)

[**Table A9: Robustness check: main treatment effect deploying students’ GPA in the last semester—instead of their admission scores—as baseline ability measure 12**](#_Toc81401699)

# **Table A4.: Treatment effect on students’ endline exam grades, among those who answered the endline questionnaire**

|  | (1) | (2) | (3) | (4) | (5) | (6) | (7) | (8) | (9) | (10) | (11) | (12) |
| --- | --- | --- | --- | --- | --- | --- | --- | --- | --- | --- | --- | --- |
| $\boldsymbol{\beta}_{\mathbf{1}}$**: Treated [T]** | 0.063+ | 0.060+ | 0.056 | 0.077 | 0.095 | 0.060 | 0.014 | 0.070 | 0.067 | 0.076 | 0.068 | -0.010 |
| *(treated = 1)* | (0.034) | (0.034) | (0.039) | (0.047) | (0.126) | (0.037) | (0.040) | (0.069) | (0.068) | (0.068) | (0.069) | (0.090) |
| $\boldsymbol{\beta}_{\mathbf{2}}$**: Exam [E]**  *(second = 1)* | -0.141*** | -0.140*** | -0.141*** | -0.141*** | -0.141*** | -0.001 | -0.134*** | -0.107 | -0.105 | -0.091 | -0.109 | -0.105 |
|  | (0.039) | (0.039) | (0.039) | (0.039) | (0.039) | (0.041) | (0.039) | (0.080) | (0.080) | (0.080) | (0.080) | (0.080) |
| $\boldsymbol{\beta}_{\mathbf{3}}$**: Carry-over [T×E]** | -0.083 | -0.084 | -0.084 | -0.083 | -0.084 | -0.070 | -0.096 | -0.023 | -0.016 | -0.038 | -0.019 | -0.022 |
|  | (0.059) | (0.059) | (0.059) | (0.059) | (0.059) | (0.062) | (0.059) | (0.121) | (0.121) | (0.120) | (0.121) | (0.121) |
| $\boldsymbol{\beta}_{\mathbf{6}}$**: Interaction^a^** |  | 0.077* | 0.021 | -0.024 | -0.033 | -0.000 | 0.267* |  | 0.072 | -0.027 | 0.031 | 0.143 |
| (T×Main effet[Z]) |  | (0.032) | (0.057) | (0.054) | (0.127) | (0.002) | (0.111) |  | (0.053) | (0.053) | (0.053) | (0.106) |
| $\boldsymbol{\beta}_{\mathbf{5}}$**:Main effects**[Z] |  |  |  |  |  |  |  |  |  |  |  |  |
| Baseline test anxiety^b^ |  |  |  |  |  |  |  | ✓ | -0.155*** | ✓ | ✓ | ✓ |
|  |  |  |  |  |  |  |  |  | (0.039) |  |  |  |
| Baseline self-confidence^b^ |  |  |  |  |  |  |  | ✓ | ✓ | 0.170*** | ✓ | ✓ |
|  |  |  |  |  |  |  |  |  |  | (0.039) |  |  |
| Baseline external control^b^ |  |  |  |  |  |  |  | ✓ | ✓ | ✓ | -0.054 | ✓ |
|  |  |  |  |  |  |  |  |  |  |  | (0.039) |  |
| Parental education |  |  |  |  |  |  |  | ✓ | ✓ | ✓ | ✓ | -0.043 |
|  |  |  |  |  |  |  |  |  |  |  |  | (0.080) |
| Students’ ability^b^ | ✓ | 0.222*** | ✓ | ✓ | ✓ | ✓ | ✓ | ✓ | ✓ | ✓ | ✓ | ✓ |
|  |  | (0.026) |  |  |  |  |  |  |  |  |  |  |
| First-year student | ✓ | ✓ | -0.085* | ✓ | ✓ | ✓ | ✓ | ✓ | ✓ | ✓ | ✓ | ✓ |
|  |  |  | (0.043) |  |  |  |  |  |  |  |  |  |
| Female | ✓ | ✓ | ✓ | 0.142*** | ✓ | ✓ | ✓ | ✓ | ✓ | ✓ | ✓ | ✓ |
|  |  |  |  | (0.042) |  |  |  |  |  |  |  |  |
| Has mobile phone |  |  |  |  | 0.058 |  |  |  |  |  |  |  |
|  |  |  |  |  | (0.092) |  |  |  |  |  |  |  |
| Day of message |  |  |  |  |  | -0.018*** |  |  |  |  |  |  |
|  |  |  |  |  |  | (0.002) |  |  |  |  |  |  |
| Exam difficulty | ✓ | ✓ | ✓ | ✓ | ✓ | ✓ | -1.904*** | ✓ | ✓ | ✓ | ✓ | ✓ |
|  |  |  |  |  |  |  | (0.086) |  |  |  |  |  |
| **Constant** | 3.140*** | 3.161*** | 3.142*** | 3.138*** | 3.090*** | 3.018*** | 3.166*** | 2.651+ | 2.771+ | 2.627+ | 2.666+ | 2.626+ |
|  | (0.724) | (0.724) | (0.724) | (0.724) | (0.729) | (0.717) | (0.724) | (1.474) | (1.466) | (1.461) | (1.474) | (1.474) |
| Observations | 8,158 | 8,158 | 8,158 | 8,158 | 8,158 | 8,158 | 8,158 | 1,981 | 1,981 | 1,981 | 1,981 | 1,981 |
| N of students | 6,809 | 6,809 | 6,809 | 6,809 | 6,809 | 6,809 | 6,809 | 1,565 | 1,565 | 1,565 | 1,565 | 1,565 |
| Cohen’s *d* effect size of $\boldsymbol{\beta}_{\mathbf{1}}$ | 0.044 | 0.042 | 0.039 | 0.054 | 0.066 | 0.042 | 0.010 | 0.051 | 0.049 | 0.055 | 0.049 | -0.008 |
| The joint linear effect of $\boldsymbol{\beta}_{\mathbf{1}}$ & $\boldsymbol{\beta}_{\mathbf{3}}$ | -0.020 | -0.024 | -0.028 | -0.006 | 0.011 | -0.011 | -0.081 | 0.047 | 0.051 | 0.038 | 0.048 | -0.032 |
|  | (0.046) | (0.046) | (0.050) | (0.056) | (0.130) | (0.057) | (0.053) | (0.094) | (0.094) | (0.094) | (0.094) | (0.111) |

All models (Column 1-12) contain the following preregistered standard baseline control variables: student’s gender, age, ability, student is a first-year student, the type of training, the financial form of training, the level of training, the difficulty of the exam, and study program fixed effects.

The table lists those variables that we preregistered as a variable to test treatment heterogeneity (Z). Some of the standard control variables are listed in the table as they appear among variables in Z. We marked these variables with the ✓ sign indicating that the given variable was included in the regression even though its estimated coefficient was not included in the table.

In addition to the standard baseline variables, columns 8-12 contain the following preregistered additional baseline variables from the baseline survey, and thus they are available for a subset of students: baseline test anxiety, baseline self-confidence, baseline external control, and parental education. Since all of the additionally used control variables were preregistered as a variable to test treatment heterogeneity (Z), all of them are listed in the table and therefore marked with the ✓ sign.

^a^ To enhance readability, the *Interaction* (T×Z) refers to the product of the treatment variable (T) and a specific main effect (Z). The coefficient of the corresponding main effect is shown in the table. For example, in Column 2, the interaction refers to the product of T×Students’ ability, and in Column 10, the *Interaction* refers to the product of T× Baseline self-confidence.

^b^ z-standardized variable at 0 mean and 1 standard deviation

Standard errors in parentheses, *** p<0.001, ** p<0.01, * p<0.05, + p<0.1

# **Table A5.: Treatment effect on students’ endline exam grades, among students who have two endline exam-grades**

|  | (1) | (2) | (3) | (4) | (5) | (6) | (7) | (8) | (9) | (10) | (11) | (12) |
| --- | --- | --- | --- | --- | --- | --- | --- | --- | --- | --- | --- | --- |
| $\boldsymbol{\beta}_{\mathbf{1}}$**: Treated [T]** | 0.005 | 0.005 | 0.000 | 0.027 | -0.015 | 0.010 | -0.010 | 0.043 | 0.045 | 0.047 | 0.046 | 0.020 |
| *(treated = 1)* | (0.021) | (0.021) | (0.024) | (0.027) | (0.066) | (0.022) | (0.024) | (0.054) | (0.054) | (0.053) | (0.054) | (0.066) |
| $\boldsymbol{\beta}_{\mathbf{2}}$**: Exam [E]**  *(second = 1)* | -0.079*** | -0.079*** | -0.079*** | -0.078*** | -0.079*** | 0.070** | -0.077*** | -0.024 | -0.022 | -0.019 | -0.020 | -0.024 |
|  | (0.022) | (0.022) | (0.022) | (0.022) | (0.022) | (0.023) | (0.022) | (0.054) | (0.054) | (0.053) | (0.054) | (0.054) |
| $\boldsymbol{\beta}_{\mathbf{3}}$**: Carry-over [T×E]** | -0.026 | -0.026 | -0.026 | -0.026 | -0.026 | -0.018 | -0.029 | -0.073 | -0.077 | -0.079 | -0.079 | -0.071 |
|  | (0.033) | (0.033) | (0.033) | (0.033) | (0.033) | (0.033) | (0.033) | (0.081) | (0.081) | (0.080) | (0.081) | (0.081) |
| $\boldsymbol{\beta}_{\mathbf{6}}$**: Interaction^a^** |  | 0.032+ | 0.016 | -0.037 | 0.021 | -0.001 | 0.088 |  | 0.001 | -0.010 | 0.016 | 0.042 |
| (T×Main effet[Z]) |  | (0.016) | (0.030) | (0.028) | (0.065) | (0.001) | (0.060) |  | (0.035) | (0.035) | (0.035) | (0.070) |
| $\boldsymbol{\beta}_{\mathbf{5}}$**:Main effects**[Z] |  |  |  |  |  |  |  |  |  |  |  |  |
| Baseline test anxiety^b^ |  |  |  |  |  |  |  | ✓ | -0.070* | ✓ | ✓ | ✓ |
|  |  |  |  |  |  |  |  |  | (0.028) |  |  |  |
| Baseline self-confidence^b^ |  |  |  |  |  |  |  | ✓ | ✓ | 0.167*** | ✓ | ✓ |
|  |  |  |  |  |  |  |  |  |  | (0.027) |  |  |
| Baseline external control^b^ |  |  |  |  |  |  |  | ✓ | ✓ | ✓ | -0.069* | ✓ |
|  |  |  |  |  |  |  |  |  |  |  | (0.027) |  |
| Parental education |  |  |  |  |  |  |  | ✓ | ✓ | ✓ | ✓ | 0.019 |
|  |  |  |  |  |  |  |  |  |  |  |  | (0.056) |
| Students’ ability^b^ | ✓ | 0.214*** | ✓ | ✓ | ✓ | ✓ | ✓ | ✓ | ✓ | ✓ | ✓ | ✓ |
|  |  | (0.015) |  |  |  |  |  |  |  |  |  |  |
| First-year student | ✓ | ✓ | -0.094*** | ✓ | ✓ | ✓ | ✓ | ✓ | ✓ | ✓ | ✓ | ✓ |
|  |  |  | (0.025) |  |  |  |  |  |  |  |  |  |
| Female | ✓ | ✓ | ✓ | 0.178*** | ✓ | ✓ | ✓ | ✓ | ✓ | ✓ | ✓ | ✓ |
|  |  |  |  | (0.024) |  |  |  |  |  |  |  |  |
| Has mobile phone |  |  |  |  | -0.011 |  |  |  |  |  |  |  |
|  |  |  |  |  | (0.051) |  |  |  |  |  |  |  |
| Day of message |  |  |  |  |  | -0.020*** |  |  |  |  |  |  |
|  |  |  |  |  |  | (0.001) |  |  |  |  |  |  |
| Exam difficulty | ✓ | ✓ | ✓ | ✓ | ✓ | ✓ | -1.652*** | ✓ | ✓ | ✓ | ✓ | ✓ |
|  |  |  |  |  |  |  | (0.048) |  |  |  |  |  |
| **Constant** | 3.743*** | 3.748*** | 3.745*** | 3.732*** | 3.753*** | 3.564*** | 3.746*** | 2.244* | 2.259* | 2.292* | 2.304* | 2.197* |
|  | (0.356) | (0.356) | (0.356) | (0.356) | (0.359) | (0.349) | (0.356) | (1.086) | (1.085) | (1.076) | (1.085) | (1.087) |
| Observations | 26,981 | 26,981 | 26,981 | 26,981 | 26,981 | 26,981 | 26,981 | 4,218 | 4,218 | 4,218 | 4,218 | 4,218 |
| N of students | 14,089 | 14,089 | 14,089 | 14,089 | 14,089 | 14,089 | 14,089 | 2,178 | 2,178 | 2,178 | 2,178 | 2,178 |
| Cohen’s *d* effect size of $\boldsymbol{\beta}_{\mathbf{1}}$ | 0.004 | 0.003 | 0.000 | 0.018 | -0.010 | 0.007 | -0.007 | 0.031 | 0.032 | 0.034 | 0.033 | 0.014 |
| The joint linear effect of $\boldsymbol{\beta}_{\mathbf{1}}$ & $\boldsymbol{\beta}_{\mathbf{3}}$ | -0.021 | -0.021 | -0.026 | 0.001 | -0.040 | -0.008 | -0.039 | -0.030 | -0.032 | -0.032 | -0.033 | -0.051 |
|  | (0.021) | (0.021) | (0.024) | (0.027) | (0.066) | (0.027) | (0.025) | (0.054) | (0.054) | (0.053) | (0.054) | (0.066) |

All models (Column 1-12) contain the following preregistered standard baseline control variables: student’s gender, age, ability, student is a first-year student, the type of training, the financial form of training, the level of training, the difficulty of the exam, and study program fixed effects.

The table lists those variables that we preregistered as a variable to test treatment heterogeneity (Z). Some of the standard control variables are listed in the table as they appear among variables in Z. We marked these variables with the ✓ sign indicating that the given variable was included in the regression even though its estimated coefficient was not included in the table.

In addition to the standard baseline variables, columns 8-12 contain the following preregistered additional baseline variables from the baseline survey, and thus they are available for a subset of students: baseline test anxiety, baseline self-confidence, baseline external control, and parental education. Since all of the additionally used control variables were preregistered as a variable to test treatment heterogeneity (Z), all of them are listed in the table and therefore marked with the ✓ sign.

^a^ To enhance readability, the *Interaction* (T×Z) refers to the product of the treatment variable (T) and a specific main effect (Z). The coefficient of the corresponding main effect is shown in the table. For example, in Column 2, the interaction refers to the product of T×Students’ ability, and in Column 10, the *Interaction* refers to the product of T× Baseline self-confidence.

^b^ z-standardized variable at 0 mean and 1 standard deviation

Standard errors in parentheses, *** p<0.001, ** p<0.01, * p<0.05, + p<0.1

# **Table A6.: Treatment effect on students’ endline test anxiety among students who answered the endline survey twice**

|  | (1) | (2) | (3) | (4) | (5) | (6) | (7) | (8) | (9) | (10) | (11) | (12) |
| --- | --- | --- | --- | --- | --- | --- | --- | --- | --- | --- | --- | --- |
| $\boldsymbol{\beta}_{\mathbf{1}}$**: Treated [T]** | -0.045 | -0.044 | 0.017 | -0.011 | -0.325 | 0.022 | -0.049 | -0.130 | -0.150 | -0.181 | -0.131 | -0.273 |
| *(treated = 1)* | (0.078) | (0.078) | (0.087) | (0.103) | (0.254) | (0.083) | (0.089) | (0.158) | (0.144) | (0.154) | (0.158) | (0.198) |
| $\boldsymbol{\beta}_{\mathbf{2}}$**: Exam [E]**  *(second = 1)* | -0.057 | -0.058 | -0.058 | -0.057 | -0.059 | -0.149 | -0.057 | -0.179 | -0.254 | -0.272 | -0.177 | -0.176 |
|  | (0.085) | (0.085) | (0.085) | (0.085) | (0.085) | (0.093) | (0.085) | (0.178) | (0.164) | (0.174) | (0.178) | (0.178) |
| $\boldsymbol{\beta}_{\mathbf{3}}$**: Carry-over [T×E]** | 0.086 | 0.086 | 0.090 | 0.085 | 0.085 | 0.203 | 0.085 | 0.312 | 0.320 | 0.406 | 0.310 | 0.310 |
|  | (0.137) | (0.137) | (0.137) | (0.137) | (0.137) | (0.146) | (0.138) | (0.290) | (0.260) | (0.282) | (0.290) | (0.290) |
| $\boldsymbol{\beta}_{\mathbf{6}}$**: Interaction^a^** |  | -0.009 | -0.186 | -0.055 | 0.295 | -0.013* | 0.021 |  | -0.123 | 0.275* | -0.124 | 0.262 |
| (T×Main effet[Z]) |  | (0.065) | (0.116) | (0.112) | (0.254) | (0.005) | (0.234) |  | (0.104) | (0.108) | (0.108) | (0.216) |
| $\boldsymbol{\beta}_{\mathbf{5}}$**:Main effects**[Z] |  |  |  |  |  |  |  |  |  |  |  |  |
| Baseline test anxiety^b^ |  |  |  |  |  |  |  | ✓ | 1.355*** | ✓ | ✓ | ✓ |
|  |  |  |  |  |  |  |  |  | (0.082) |  |  |  |
| Baseline self-confidence^b^ |  |  |  |  |  |  |  | ✓ | ✓ | -0.821*** | ✓ | ✓ |
|  |  |  |  |  |  |  |  |  |  | (0.087) |  |  |
| Baseline external control^b^ |  |  |  |  |  |  |  | ✓ | ✓ | ✓ | 0.075 | ✓ |
|  |  |  |  |  |  |  |  |  |  |  | (0.087) |  |
| Parental education |  |  |  |  |  |  |  | ✓ | ✓ | ✓ | ✓ | -0.211 |
|  |  |  |  |  |  |  |  |  |  |  |  | (0.181) |
| Students’ ability^b^ | ✓ | -0.052 | ✓ | ✓ | ✓ | ✓ | ✓ | ✓ | ✓ | ✓ | ✓ | ✓ |
|  |  | (0.058) |  |  |  |  |  |  |  |  |  |  |
| First-year student | ✓ | ✓ | 0.171+ | ✓ | ✓ | ✓ | ✓ | ✓ | ✓ | ✓ | ✓ | ✓ |
|  |  |  | (0.096) |  |  |  |  |  |  |  |  |  |
| Female | ✓ | ✓ | ✓ | 1.151*** | ✓ | ✓ | ✓ | ✓ | ✓ | ✓ | ✓ | ✓ |
|  |  |  |  | (0.093) |  |  |  |  |  |  |  |  |
| Has mobile phone |  |  |  |  | -0.164 |  |  |  |  |  |  |  |
|  |  |  |  |  | (0.202) |  |  |  |  |  |  |  |
| Day of message |  |  |  |  |  | 0.010* |  |  |  |  |  |  |
|  |  |  |  |  |  | (0.004) |  |  |  |  |  |  |
| Exam difficulty | ✓ | ✓ | ✓ | ✓ | ✓ | ✓ | 1.070*** | ✓ | ✓ | ✓ | ✓ | ✓ |
|  |  |  |  |  |  |  | (0.185) |  |  |  |  |  |
| **Constant** | 7.842*** | 7.840*** | 7.809*** | 7.840*** | 8.014*** | 7.848*** | 7.844*** | 10.477** | 9.097** | 10.652** | 10.585** | 10.531** |
|  | (1.611) | (1.611) | (1.611) | (1.611) | (1.621) | (1.610) | (1.611) | (3.497) | (3.154) | (3.396) | (3.499) | (3.499) |
| Observations | 7,922 | 7,922 | 7,922 | 7,922 | 7,922 | 7,922 | 7,922 | 1,947 | 1,947 | 1,947 | 1,947 | 1,947 |
| N of students | 6,531 | 6,531 | 6,531 | 6,531 | 6,531 | 6,531 | 6,531 | 1,523 | 1,523 | 1,523 | 1,523 | 1,523 |
| Cohen’s *d* effect size of $\boldsymbol{\beta}_{\mathbf{1}}$ | -0.015 | -0.015 | -0.015 | -0.015 | -0.015 | -0.015 | -0.015 | -0.015 | -0.015 | -0.015 | -0.015 | -0.015 |
| The joint linear effect of $\boldsymbol{\beta}_{\mathbf{1}}$ & $\boldsymbol{\beta}_{\mathbf{3}}$ | 0.041 | 0.042 | 0.107 | 0.074 | -0.240 | 0.225 | 0.036 | 0.182 | 0.170 | 0.225 | 0.179 | 0.036 |
|  | (0.099) | (0.099) | (0.107) | (0.118) | (0.262) | (0.126) | (0.113) | (0.205) | (0.190) | (0.201) | (0.205) | (0.238) |

All models (Column 1-12) contain the following preregistered standard baseline control variables: student’s gender, age, ability, student is a first-year student, the type of training, the financial form of training, the level of training, the difficulty of the exam, and study program fixed effects.

The table lists those variables that we preregistered as a variable to test treatment heterogeneity (Z). Some of the standard control variables are listed in the table as they appear among variables in Z. We marked these variables with the ✓ sign indicating that the given variable was included in the regression even though its estimated coefficient was not included in the table.

In addition to the standard baseline variables, columns 8-12 contain the following preregistered additional baseline variables from the baseline survey, and thus they are available for a subset of students: baseline test anxiety, baseline self-confidence, baseline external control, and parental education. Since all of the additionally used control variables were preregistered as a variable to test treatment heterogeneity (Z), all of them are listed in the table and therefore marked with the ✓ sign.

^a^ To enhance readability, the *Interaction* (T×Z) refers to the product of the treatment variable (T) and a specific main effect (Z). The coefficient of the corresponding main effect is shown in the table. For example, in Column 2, the interaction refers to the product of T×Students’ ability, and in Column 10, the *Interaction* refers to the product of T× Baseline self-confidence.

^b^ z-standardized variable at 0 mean and 1 standard deviation

Standard errors in parentheses, *** p<0.001, ** p<0.01, * p<0.05, + p<0.1

# **Table A7.: Treatment effect on students’ endline self-efficacy among students who answered the endline survey twice**

|  | (1) | (2) | (3) | (4) | (5) | (6) | (7) | (8) | (9) | (10) | (11) | (12) |
| --- | --- | --- | --- | --- | --- | --- | --- | --- | --- | --- | --- | --- |
| $\boldsymbol{\beta}_{\mathbf{1}}$**: Treated [T]** | 0.273*** | 0.275*** | 0.313*** | 0.260** | 0.558* | 0.255*** | 0.197* | 0.288* | 0.299* | 0.342** | 0.277* | 0.221 |
| *(treated = 1)* | (0.068) | (0.068) | (0.075) | (0.089) | (0.221) | (0.072) | (0.077) | (0.136) | (0.130) | (0.126) | (0.135) | (0.169) |
| $\boldsymbol{\beta}_{\mathbf{2}}$**: Exam [E]**  *(second = 1)* | -0.214** | -0.214** | -0.214** | -0.214** | -0.212** | 0.001 | -0.201** | -0.103 | -0.069 | -0.009 | -0.107 | -0.099 |
|  | (0.074) | (0.074) | (0.074) | (0.074) | (0.074) | (0.080) | (0.074) | (0.153) | (0.148) | (0.143) | (0.152) | (0.153) |
| $\boldsymbol{\beta}_{\mathbf{3}}$**: Carry-over [T×E]** | 0.013 | 0.014 | 0.016 | 0.013 | 0.014 | 0.005 | -0.009 | -0.027 | -0.024 | -0.121 | -0.014 | -0.032 |
|  | (0.118) | (0.118) | (0.118) | (0.118) | (0.118) | (0.126) | (0.119) | (0.249) | (0.238) | (0.229) | (0.247) | (0.249) |
| $\boldsymbol{\beta}_{\mathbf{6}}$**: Interaction^a^** |  | -0.046 | -0.121 | 0.023 | -0.300 | 0.003 | 0.408* | 0.227* | -0.175* | 0.112 | 0.129 | 0.227* |
| (T×Main effet[Z]) |  | (0.057) | (0.101) | (0.097) | (0.221) | (0.005) | (0.204) | (0.090) | (0.089) | (0.092) | (0.184) | (0.090) |
| $\boldsymbol{\beta}_{\mathbf{5}}$**:Main effects**[Z] |  |  |  |  |  |  |  |  |  |  |  |  |
| Baseline test anxiety^b^ |  |  |  |  |  |  |  | ✓ | -0.849*** | ✓ | ✓ | ✓ |
|  |  |  |  |  |  |  |  |  | (0.074) |  |  |  |
| Baseline self-confidence^b^ |  |  |  |  |  |  |  | ✓ | ✓ | 1.090*** | ✓ | ✓ |
|  |  |  |  |  |  |  |  |  |  | (0.071) |  |  |
| Baseline external control^b^ |  |  |  |  |  |  |  | ✓ | ✓ | ✓ | -0.333*** | ✓ |
|  |  |  |  |  |  |  |  |  |  |  | (0.074) |  |
| Parental education |  |  |  |  |  |  |  | ✓ | ✓ | ✓ | ✓ | -0.197 |
|  |  |  |  |  |  |  |  |  |  |  |  | (0.155) |
| Students’ ability^b^ | ✓ | 0.094+ | ✓ | ✓ | ✓ | ✓ | ✓ | ✓ | ✓ | ✓ | ✓ | ✓ |
|  |  | (0.050) |  |  |  |  |  |  |  |  |  |  |
| First-year student | ✓ | ✓ | 0.150+ | ✓ | ✓ | ✓ | ✓ | ✓ | ✓ | ✓ | ✓ | ✓ |
|  |  |  | (0.083) |  |  |  |  |  |  |  |  |  |
| Female | ✓ | ✓ | ✓ | -0.454*** | ✓ | ✓ | ✓ | ✓ | ✓ | ✓ | ✓ | ✓ |
|  |  |  |  | (0.081) |  |  |  |  |  |  |  |  |
| Has mobile phone |  |  |  |  | 0.184 |  |  |  |  |  |  |  |
|  |  |  |  |  | (0.175) |  |  |  |  |  |  |  |
| Day of message |  |  |  |  |  | -0.025*** |  |  |  |  |  |  |
|  |  |  |  |  |  | (0.004) |  |  |  |  |  |  |
| Exam difficulty | ✓ | ✓ | ✓ | ✓ | ✓ | ✓ | -1.639*** | ✓ | ✓ | ✓ | ✓ | ✓ |
|  |  |  |  |  |  |  | (0.160) |  |  |  |  |  |
| **Constant** | 7.600*** | 7.592*** | 7.585*** | 7.602*** | 7.413*** | 7.449*** | 7.634*** | 6.891* | 7.741** | 6.735* | 7.078* | 7.021* |
|  | (1.401) | (1.401) | (1.401) | (1.401) | (1.409) | (1.394) | (1.400) | (2.984) | (2.848) | (2.740) | (2.964) | (2.986) |
| Observations | 7,906 | 7,906 | 7,906 | 7,906 | 7,906 | 7,906 | 7,906 | 1,950 | 1,950 | 1,950 | 1,950 | 1,950 |
| N of students | 6,518 | 6,518 | 6,518 | 6,518 | 6,518 | 6,518 | 6,518 | 1,528 | 1,528 | 1,528 | 1,528 | 1,528 |
| Cohen’s *d* effect size of $\boldsymbol{\beta}_{\mathbf{1}}$ | 0.108 | 0.109 | 0.124 | 0.103 | 0.221 | 0.101 | 0.078 | 0.112 | 0.117 | 0.134 | 0.108 | 0.086 |
| The joint linear effect of $\boldsymbol{\beta}_{\mathbf{1}}$ & $\boldsymbol{\beta}_{\mathbf{3}}$ | 0.286** | 0.289** | 0.329*** | 0.273** | 0.572* | 0.260* | 0.189+ | 0.261 | 0.275 | 0.222 | 0.263 | 0.189 |
|  | (0.085) | (0.086) | (0.093) | (0.103) | (0.228) | (0.109) | (0.098) | (0.176) | (0.170) | (0.165) | (0.175) | (0.203) |

All models (Column 1-12) contain the following preregistered standard baseline control variables: student’s gender, age, ability, student is a first-year student, the type of training, the financial form of training, the level of training, the difficulty of the exam, and study program fixed effects.

The table lists those variables that we preregistered as a variable to test treatment heterogeneity (Z). Some of the standard control variables are listed in the table as they appear among variables in Z. We marked these variables with the ✓ sign indicating that the given variable was included in the regression even though its estimated coefficient was not included in the table.

In addition to the standard baseline variables, columns 8-12 contain the following preregistered additional baseline variables from the baseline survey, and thus they are available for a subset of students: baseline test anxiety, baseline self-confidence, baseline external control, and parental education. Since all of the additionally used control variables were preregistered as a variable to test treatment heterogeneity (Z), all of them are listed in the table and therefore marked with the ✓ sign.

^a^ To enhance readability, the *Interaction* (T×Z) refers to the product of the treatment variable (T) and a specific main effect (Z). The coefficient of the corresponding main effect is shown in the table. For example, in Column 2, the interaction refers to the product of T×Students’ ability, and in Column 10, the *Interaction* refers to the product of T× Baseline self-confidence.

^b^ z-standardized variable at 0 mean and 1 standard deviation

Standard errors in parentheses, *** p<0.001, ** p<0.01, * p<0.05, + p<0.1

# **Table A8.: Treatment effect on students’ endline motivation to do well on the exam among students who answered the endline survey twice**

|  | (1) | (2) | (3) | (4) | (5) | (6) | (7) | (8) | (9) | (10) | (11) | (12) |
| --- | --- | --- | --- | --- | --- | --- | --- | --- | --- | --- | --- | --- |
| $\boldsymbol{\beta}_{\mathbf{1}}$**: Treated [T]** | 0.082+ | 0.082+ | 0.136** | 0.072 | 0.198 | 0.084+ | 0.057 | 0.055 | 0.056 | 0.061 | 0.053 | 0.168+ |
| *(treated = 1)* | (0.042) | (0.042) | (0.047) | (0.056) | (0.140) | (0.045) | (0.048) | (0.080) | (0.080) | (0.080) | (0.080) | (0.101) |
| $\boldsymbol{\beta}_{\mathbf{2}}$**: Exam [E]**  *(second = 1)* | -0.009 | -0.009 | -0.010 | -0.009 | -0.009 | 0.041 | -0.005 | 0.035 | 0.034 | 0.045 | 0.034 | 0.035 |
|  | (0.046) | (0.046) | (0.046) | (0.046) | (0.046) | (0.050) | (0.046) | (0.091) | (0.091) | (0.091) | (0.091) | (0.091) |
| $\boldsymbol{\beta}_{\mathbf{3}}$**: Carry-over [T×E]** | -0.097 | -0.097 | -0.094 | -0.097 | -0.097 | -0.089 | -0.104 | -0.121 | -0.122 | -0.132 | -0.119 | -0.127 |
|  | (0.074) | (0.074) | (0.074) | (0.074) | (0.074) | (0.079) | (0.074) | (0.147) | (0.147) | (0.146) | (0.147) | (0.147) |
| $\boldsymbol{\beta}_{\mathbf{6}}$**: Interaction^a^** |  | 0.011 | -0.162* | 0.017 | -0.122 | -0.001 | 0.135 |  | 0.038 | -0.008 | 0.002 | -0.194+ |
| (T×Main effet[Z]) |  | (0.036) | (0.064) | (0.061) | (0.140) | (0.003) | (0.128) |  | (0.055) | (0.056) | (0.055) | (0.111) |
| $\boldsymbol{\beta}_{\mathbf{5}}$**:Main effects**[Z] |  |  |  |  |  |  |  |  |  |  |  |  |
| Baseline test anxiety^b^ |  |  |  |  |  |  |  | ✓ | -0.006 | ✓ | ✓ | ✓ |
|  |  |  |  |  |  |  |  |  | (0.046) |  |  |  |
| Baseline self-confidence^b^ |  |  |  |  |  |  |  | ✓ | ✓ | 0.117** | ✓ | ✓ |
|  |  |  |  |  |  |  |  |  |  | (0.045) |  |  |
| Baseline external control^b^ |  |  |  |  |  |  |  | ✓ | ✓ | ✓ | -0.060 | ✓ |
|  |  |  |  |  |  |  |  |  |  |  | (0.044) |  |
| Parental education |  |  |  |  |  |  |  | ✓ | ✓ | ✓ | ✓ | -0.072 |
|  |  |  |  |  |  |  |  |  |  |  |  | (0.092) |
| Students’ ability^b^ | ✓ | -0.006 | ✓ | ✓ | ✓ | ✓ | ✓ | ✓ | ✓ | ✓ | ✓ | ✓ |
|  |  | (0.031) |  |  |  |  |  |  |  |  |  |  |
| First-year student | ✓ | ✓ | 0.010 | ✓ | ✓ | ✓ | ✓ | ✓ | ✓ | ✓ | ✓ | ✓ |
|  |  |  | (0.052) |  |  |  |  |  |  |  |  |  |
| Female | ✓ | ✓ | ✓ | 0.145** | ✓ | ✓ | ✓ | ✓ | ✓ | ✓ | ✓ | ✓ |
|  |  |  |  | (0.050) |  |  |  |  |  |  |  |  |
| Has mobile phone |  |  |  |  | 0.046 |  |  |  |  |  |  |  |
|  |  |  |  |  | (0.109) |  |  |  |  |  |  |  |
| Day of message |  |  |  |  |  | -0.006** |  |  |  |  |  |  |
|  |  |  |  |  |  | (0.002) |  |  |  |  |  |  |
| Exam difficulty | ✓ | ✓ | ✓ | ✓ | ✓ | ✓ | -0.183+ | ✓ | ✓ | ✓ | ✓ | ✓ |
|  |  |  |  |  |  |  | (0.100) |  |  |  |  |  |
| **Constant** | 9.334*** | 9.336*** | 9.294*** | 9.336*** | 9.280*** | 9.265*** | 9.341*** | 14.952*** | 15.033*** | 14.593*** | 14.990*** | 14.861*** |
|  | (0.974) | (0.974) | (0.974) | (0.974) | (0.979) | (0.973) | (0.974) | (1.958) | (1.961) | (1.957) | (1.956) | (1.956) |
| Observations | 7,910 | 7,910 | 7,910 | 7,910 | 7,910 | 7,910 | 7,910 | 1,950 | 1,950 | 1,950 | 1,950 | 1,950 |
| N of students | 6,525 | 6,525 | 6,525 | 6,525 | 6,525 | 6,525 | 6,525 | 1,526 | 1,526 | 1,526 | 1,526 | 1,526 |
| Cohen’s *d* effect size of $\boldsymbol{\beta}_{\mathbf{1}}$ | 0.054 | 0.053 | 0.089 | 0.047 | 0.129 | 0.055 | 0.037 | 0.037 | 0.038 | 0.041 | 0.036 | 0.113 |
| The joint linear effect of $\boldsymbol{\beta}_{\mathbf{1}}$ & $\boldsymbol{\beta}_{\mathbf{3}}$ | -0.015 | -0.016 | 0.042 | -0.025 | 0.101 | -0.005 | -0.047 | -0.065 | -0.066 | -0.071 | -0.066 | 0.040 |
|  | (0.054) | (0.054) | (0.058) | (0.065) | (0.144) | (0.069) | (0.062) | (0.105) | (0.105) | (0.105) | (0.105) | (0.121) |

All models (Column 1-12) contain the following preregistered standard baseline control variables: student’s gender, age, ability, student is a first-year student, the type of training, the financial form of training, the level of training, the difficulty of the exam, and study program fixed effects.

The table lists those variables that we preregistered as a variable to test treatment heterogeneity (Z). Some of the standard control variables are listed in the table as they appear among variables in Z. We marked these variables with the ✓ sign indicating that the given variable was included in the regression even though its estimated coefficient was not included in the table.

In addition to the standard baseline variables, columns 8-12 contain the following preregistered additional baseline variables from the baseline survey, and thus they are available for a subset of students: baseline test anxiety, baseline self-confidence, baseline external control, and parental education. Since all of the additionally used control variables were preregistered as a variable to test treatment heterogeneity (Z), all of them are listed in the table and therefore marked with the ✓ sign.

^a^ To enhance readability, the *Interaction* (T×Z) refers to the product of the treatment variable (T) and a specific main effect (Z). The coefficient of the corresponding main effect is shown in the table. For example, in Column 2, the interaction refers to the product of T×Students’ ability, and in Column 10, the *Interaction* refers to the product of T× Baseline self-confidence.

^b^ z-standardized variable at 0 mean and 1 standard deviation

Standard errors in parentheses, *** p<0.001, ** p<0.01, * p<0.05, + p<0.1

# **Table A9: Robustness check: main treatment effect deploying students’ GPA in the last semester—instead of their admission scores—as baseline ability measure**

|  | Exam grades | Test anxiety | Self-efficacy | Motivation to do well on the exam |
| --- | --- | --- | --- | --- |
| $\boldsymbol{\beta}_{\mathbf{1}}$**: Treated [T]** | 0.008 | -0.047 | 0.295*** | 0.101* |
| *(treated = 1)* | (0.020) | (0.075) | (0.065) | (0.041) |
| $\boldsymbol{\beta}_{\mathbf{2}}$**: Exam [E]**  *(second = 1)* | -0.083*** | -0.069 | -0.190** | 0.002 |
|  | (0.021) | (0.084) | (0.072) | (0.045) |
| $\boldsymbol{\beta}_{\mathbf{3}}$**: Carry-over [T×E]** | -0.024 | 0.085 | -0.021 | -0.123+ |
|  | (0.031) | (0.135) | (0.116) | (0.072) |
| Constant | 3.685*** | 7.644*** | 7.712*** | 9.163*** |
|  | (0.350) | (1.611) | (1.395) | (0.973) |
| Observations | 28,156 | 8,316 | 8,296 | 8,301 |
| Number of groups | 15,264 | 6,925 | 6,908 | 6,916 |
| Cohen’s *d* effect size | 0.01 | -0.02 | 0.12 | 0.07 |

All models contain the following preregistered standard baseline control variables: student’s gender, age, student is a first-year student, the type of training, the financial form of training, the level of training, the difficulty of the exam, and study program fixed effects.

Instead of the preregistered students’ ability we control for student’s GPA in the last semester. Standard errors in parentheses, *** p<0.001, ** p<0.01, * p<0.05, + p<0.1
