# Supplementary material for: Not just words! Effects of a light-touch randomized encouragement intervention on students’ exam grades, self-efficacy, motivation, and test anxiety
Source: PLoS One. 2021 Sep 15;16(9):e0256960. doi: 10.1371/journal.pone.0256960 (PMC8443032; doi:10.1371/journal.pone.0256960)
Supplement: S8 Appendix — (DOCX) [file pone.0256960.s008.docx]

**S8 Appendix: The original Hungarian version of various survey instruments**

The appendix belongs to the following paper by **Tamás Keller** and **Péter Szakál**:

Not just words! Effects of a light-touch randomized encouragement intervention on students’ exam grades, self-efficacy, motivation, and test anxiety

**Content of the document**

[**The original Hungarian version of the e-mail message 2**](#_Toc81399160)

[**The original Hungarian version of the SMS message 2**](#_Toc81399161)

[**The original Hungarian version of the secondary outcome variables 2**](#_Toc81399162)

[**The original Hungarian version of the baseline background questionnaire 3**](#_Toc81399163)

[**The original Hungarian version of the online survey questions about students’ experiences with our campaign 6**](#_Toc81399164)

# **The original Hungarian version of the e-mail message**

Kedves Hallgató! Az, hogy Ön hamarosan vizsgázik, annak bizonyítéka, hogy számtalan sikeres vizsga áll már Ön mögött. Őszintén bízom benne, hogy a soron következőt is sikeresen fogja teljesíteni. Ehhez sok sikert kívánok! Kérem, ezen a linken válaszoljon három egyszerű kérdésre. A válaszadók között összesen 100 ezer forint értékben sorsolunk ki az SZTE Ajándékboltba szóló utalványokat. A nyerteseket emailben értesítjük.

Az Oktatási Igazgatóság nevében Szakál Péter

# **The original Hungarian version of the SMS message**

Soron következő vizsgájához sok sikert kívánunk, hiszen eddig tanulmányai során is eredményesen bizonyította rátermettségét! SZTE Oktatási Igazgatóság

# **The original Hungarian version of the secondary outcome variables**

A következő táblázatban különböző érzéseket leíró mondatokat olvashat. Kérem, egy 0-tól 10-ig terjedő skálán jelölje be, hogy a mostani érzésit figyelembe véve Ön mennyire érzi azt, hogy az adott kijelentés megfelel annak, amit most érez. A 0 azt jelenti, hogy Ön a jelenlegi érzései alapján egyáltalán nem így érez. A 10-es azt jelenti, hogy Ön jelenlegi érzései alapján teljes mértékben így érez. A 0 és 10 közötti számokkal árnyalhatja véleményét. [1] Biztos vagyok benne, hogy holnap sikeresen fogok vizsgázni; [2] Szeretnék jól teljesíteni a holnapi vizsgámon; [3] Izgulok a holnapi vizsgám miatt.

# **The original Hungarian version of the baseline background questionnaire**

1. **Kérem, jelölje a megfelelő válasz megjelölésével, hogy az Ön esetében „Igaz” vagy „Hamis” az adott állítás!**

|  | Igaz | Hamis |
| --- | --- | --- |
| 1. Egy fontos vizsga alatt gyakran gondolok arra, hogy a többiek mennyivel okosabbak nálam. | 1 | 2 |
| 1. Vizsgák alatt nagyon izzadok. |  |  |
| 1. Vizsgák alatt gyakran gondolok olyan dolgokra, amelyek nem tartoznak szorosan a tananyaghoz. | 1 | 2 |
| 1. Fontos vizsgák alatt néha erősen ver a szívem. | 1 | 2 |
| 1. Pánikba esem, amikor váratlan vizsga helyzetbe kerülök. | 1 | 2 |
| 1. Vizsgák alatt gyakran gondolok arra, hogy mi lesz, ha megbukok. | 1 | 2 |
| 1. Vizsgák alatt gyakran annyira izgulok, hogy az sem jut eszembe, amit megtanultam. | 1 | 2 |
| 1. Még akkor is nagyon izgulok a vizsgán, ha jól felkészültem rá. | 1 | 2 |
| 1. Fontos vizsgák előtt nem tudok enni. | 1 | 2 |
| 1. Fontos vizsgák előtt gyakran remeg a kezem vagy a karom. | 1 | 2 |

1. **A lenti táblázatban különböző érzéseket és véleményeket kifejező állításokat olvashat. Olvasson el figyelmesen minden egyes állítást, és a táblázatban lévő megfelelő érték megjelölésével döntse el, hogy azzal milyen mértékben ért egyet.**

|  | Egyáltalán nem értek egyet | Inkább nem értek egyet | Inkább egyetértek | Teljes mértékben egyetértek |
| --- | --- | --- | --- | --- |
| 1. A tudományos teljesítmény olyan terület, ahol meg tudom mutatni a képességeimet és a teljesítményemért elismerést kapok. | 1 | 2 | 3 | 4 |
| 1. Gyakran gondolkodom azon, hogy vajon elég okos vagyok-e ahhoz, hogy elérjem a szakmai és tudományos céljaimat. | 1 | 2 | 3 | 4 |
| 1. Úgy gondolom, hogy évfolyamtársaimhoz képest nem vagyok elég jó tanuló. | 1 | 2 | 3 | 4 |
| 1. Zavar, hogy észbeli képességeim nem érnek fel évfolyamtársaim észbeli képességeihez. | 1 | 2 | 3 | 4 |
| 1. Ha felveszek egy új tantárgyat, akkor biztos vagyok abban, hogy a legjobb 25% között fogok végezni. | 1 | 2 | 3 | 4 |
| 1. Ha egy fontos vizsgát kell leraknom, vagy egy zárthelyi dolgozatot kell írnom, akkor tudom, hogy ez sikerülni fog. | 1 | 2 | 3 | 4 |
| 1. Keresem az intellektuális kihívásokat, mert tudom, hogy jobban fogok teljesíteni, mint a legtöbb ember. | 1 | 2 | 3 | 4 |

1. **A következő kijelentés-párok esetében válassza ki, melyik állítással ért inkább egyet!**

| **A.** | 1 – | *Ami velem történik, az elsősorban rajtam múlik.* |
| --- | --- | --- |
|  |  | **vagy** |
|  | 2 – | *Gyakran úgy érzem, nem rajtam múlik, hogyan alakul az életem.* |

| **B.** | 1 – | *Amit eltervezek, azt többnyire véghez is viszem.* |
| --- | --- | --- |
|  |  | **vagy** |
|  | 2 – | *Nem mindig bölcs dolog túl hosszú távra előre tervezni, sok minden úgyis a szerencsén múlik.* |

| **C.** | 1 – | *Abban, hogy elérem-e azt, amit akarok, alig van szerepe a szerencsének.* |
| --- | --- | --- |
|  |  | **vagy** |
|  | 2 – | *Sokszor akár pénzfeldobással is eldönthetnénk, hogy mit tegyünk.* |

| **D.** | 1 – | *.Nem tudom elhinni, hogy a szerencse vagy a véletlen komoly szerepet játszhat az életemben.* |
| --- | --- | --- |
|  |  | **vagy** |
|  | 2 – | *Gyakran úgy érzem, alig van hatásom arra, ami velem történik* |

1. **Kérem, jelölje meg, hogy mi édesanyja (nevelőanyja) legmagasabb iskolai végzettsége?**

*Csak* ***egy választ*** *jelöljön meg!*

1. Nem fejezte be az általános iskolát 1
2. Általános iskola 2
3. Szakiskola 3
4. Szakmunkásképző 4
5. Érettségi (gimnázium vagy szakközép iskola) 5
6. Főiskola (felsőfokú alapképzés) 6
7. Egyetem (felsőfokú mesterképzés) 7
8. Nem tudom 8
9. **Kérem, jelölje meg, hogy mi édesapja (nevelőapja) legmagasabb iskolai végzettsége?**

*Csak* ***egy választ*** *jelöljön meg!*

1. Nem fejezte be az általános iskolát 1
2. Általános iskola 2
3. Szakiskola 3
4. Szakmunkásképző 4
5. Érettségi (gimnázium vagy szakközép iskola) 5
6. Főiskola (felsőfokú alapképzés) 6
7. Egyetem (felsőfokú mesterképzés) 7
8. Nem tudom 8

# **The original Hungarian version of the online survey questions about students’ experiences with our campaign**

Kedves Hallgató!

Kérjük, az alábbi rövid kérdőívet csak akkor töltse ki, ha 2019 decemberében kapott a vizsgája előtt kaptál e-mail vagy sms üzenetet a Szegedi Tudományegyetemtől.

Kérjük, támogassa a kutatásunkat azzal, hogy egy vizsgaidőszaknyi időtávlatból vissza emlékszik arra a napra, amikor az üzenet kapta

**Kérem mondja meg, hogy 2019 decemberében kapott-e a vizsgája előtt e-mail vagy sms üzenetet a Szegedi Tudományegyetemtől?** (csak egy válasz is lehetséges)

1. E-mail
2. Sms
3. Nem emlékszem

*Most gondoljon arra az üzentre, amelyet először olvasott*

**Kérem karikázza be azt, hogy emlékei szerint mi volt az üzenet tartalma?** (csak egy

válasz is lehetséges)

1. Az üzenet emlékeztetett arra, hogy vizsgám lesz.
2. Az üzenet bátorító szavakat közölt és sikeres vizsgát kívánt.
3. Az üzenet figyelmeztetett arra, hogy tanulnom kell.
4. Az üzenet figyelmeztetett arra, hogy tartozásom van a könyvtárban.

**Mennyire hitte el az üzenet tartalmát?** (csak egy válasz is lehetséges)

1. Teljes mértékben elhittem
2. Elhittem
3. El is hittem meg nem is
4. Nem hittem el
5. Egyáltalán nem hittem el

**Mennyire örült az üzenetnek?** (csak egy válasz is lehetséges)

1. Nagyon örültem
2. Örültem
3. Örültem is meg nem is
4. Nem örültem
5. Nagyon nem örültem

**Amikor Ön még nem kapott üzenetet az egyetemtől, hallott-e arról, hogy mások kaptak bátorító üzenetet az egyetemtől, amelyben az egyetem sok sikert kíván a vizsgájukhoz?** (csak egy válasz is lehetséges)

1. Igen
2. Nem
3. Nem fordult elő ilyen

**Mennyire szomorította el Önt az, hogy miközben Ön nem kapott, mások kaptak bátorító üzenetet az egyetemtől, amelyben az egyetem sok sikert kíván a vizsgájukhoz?** (csak egy válasz is lehetséges)

1. Nagyon elszomorított
2. Elszomorított
3. El is szomorított me nem is
4. Nem szomorított el
5. Egyáltalán nem szomorított el

**Ön tovább küldte-e másoknak azt a bátorító üzenetet, amelyben az egyetem sok sikert kívánt a vizsgájához?** (több válasz is lehetséges)

1. Nem osztottam meg
2. Igen megosztottam az e-mailt/sms-t az egyetemi barátaimmal
3. Igen megosztottam az e-mailt/sms-t a családommal

**Szeretne-e Ön a jövőben is hasonló bátorító üzeneteket kapni a Szegedi Tudományegyetemtől?** (csak egy válasz lehetséges)

1. Igen
2. Nem
