# Supplementary material for: Not just words! Effects of a light-touch randomized encouragement intervention on students’ exam grades, self-efficacy, motivation, and test anxiety
Source: PLoS One. 2021 Sep 15;16(9):e0256960. doi: 10.1371/journal.pone.0256960 (PMC8443032; doi:10.1371/journal.pone.0256960)
Supplement: S9 Appendix — (DOCX) [file pone.0256960.s009.docx]

**S9 Appendix: The English version of various survey instruments**

The appendix belongs to the following paper by **Tamás Keller** and **Péter Szakál**:

Not just words! Effects of a light-touch randomized encouragement intervention on students’ exam grades, self-efficacy, motivation, and test anxiety

**Content of the document**

[**The English version of the baseline background questionnaire** 2](#_Toc81399462)

[**The translated English version of the online survey questions about students’ experiences with our campaign** 6](#_Toc81399463)

# **The English version of the baseline background questionnaire**

Test Anxiety scale by Sarason [1]

1. **Please indicate whether the statement is "True" or "False" by ticking the appropriate answer.**

|  | True | False |
| --- | --- | --- |
| 1. While taking an important exam, I find myself thinking of how much brighter the other students are than I am. | 1 | 2 |
| 1. While taking an important exam, I perspire a great deal |  |  |
| 1. During class examinations, I find myself thinking of things unrelated to the actual course material | 1 | 2 |
| 1. I sometimes feel my heart beating very fast during important exams | 1 | 2 |
| 1. I get to feeling very panicky when I have to take a surprise exam | 1 | 2 |
| 1. During a test, I find myself thinking of the consequences of failing. | 1 | 2 |
| 1. During a course examination, I frequently get so nervous that I forget facts I really know. | 1 | 2 |
| 1. Even when I'm well prepared for a test, I feel very anxious about it | 1 | 2 |
| 1. I don't enjoy eating before an important test | 1 | 2 |
| 1. Before an important examination, I find my hands or arms trembling | 1 | 2 |

Academic self-confidence scale by Shrauger and Schohn [2]

1. **In the table below, you can read statements expressing different feelings and opinions. Read each statement carefully and decide to what extent you agree with it by ticking the corresponding box in the table.**

|  | Strongly disagree | Mainly disagree | Mainly agree | Strongly agree |
| --- | --- | --- | --- | --- |
| 1. Academic performance is an area in which I can show my competence and be recognized for my achievement (+) | 1 | 2 | 3 | 4 |
| 1. I frequently wonder whether I have the intellectual ability to successfully achieve my vocational and academic goals (-) | 1 | 2 | 3 | 4 |
| 1. I have recognized that I am not as good a student as most of the people with whom I am competing (-) | 1 | 2 | 3 | 4 |
| 1. It bothers me that I don't measure up to others intellectually (-) | 1 | 2 | 3 | 4 |
| 1. When I take a new course, I am usually sure that I will end up in the top 25% of the class (+) | 1 | 2 | 3 | 4 |
| 1. When I have to come through an important test or other academic assignments, I know that I can do it (+) | 1 | 2 | 3 | 4 |
| 1. I seek out activities that are intellectually challenging, because I know I can do them better than most people (+) | 1 | 2 | 3 | 4 |

+ positively worded item

– negatively worded item

Locus of control scale used by Andrisani [3]

1. **For the following pairs of statements, choose which statement you agree with more!**

| **A.** | 1 – | *What happens to me is my own doing.* |
| --- | --- | --- |
|  |  | **or** |
|  | 2 – | *Sometimes I feel that I don't have enough control over the direction my life is taking.* |

| **B.** | 1 – | *When I make plans, I am al- most certain I can make themwork.* |
| --- | --- | --- |
|  |  | **or** |
|  | 2 – | *It is not always wise to plan too far ahead, because many things turn out to be a matter of good or bad fortune anyhow.* |

| **C.** | 1 – | *In my case, getting what I want has little or nothing to do with luck.* |
| --- | --- | --- |
|  |  | **or** |
|  | 2 – | *Many times we might just as well decide what to do by flipping a coin.* |

| **D.** | 1 – | *.* *Many times I feel that I have little influence over the things that happen to me.* |
| --- | --- | --- |
|  |  | **or** |
|  | 2 – | *It is impossible for me to believe that chance or luck plays an important role in my life.* |

1. **What is your mother’s highest educational level? If you are living with your stepmother, then please indicate her highest educational level!**

*Only* ***one answer*** *is possible!*

a) Unfinished primary school 1

b) Completed primary school 2

c) Vocational school ([*Szakiskola*] new term) 3

d) Vocational school ([*Szakmunkásképző*] old term) 4

e) High-school final examination 5

f) BA, BSc 6

g) MA, MSc 7

h) Don’t know 8

1. **What is your father’s highest educational level? If you are living with your stepfather, then please indicate his highest educational level!**

*Only* ***one answer*** *is possible!*

a) Unfinished primary school 1

b) Completed primary school 2

c) Vocational school ([*Szakiskola*] new term) 3

d) Vocational school ([*Szakmunkásképző*] old term) 4

e) High-school final examination 5

f) BA, BSc 6

g) MA, MSc 7

h) Don’t know 8

# **The translated English version of the online survey questions about students’ experiences with our campaign**

Dear Student!

Please fill in the following short questionnaire only in that case if you received an e-mail or SMS message from the University of Szeged before your exam in December 2019.

Please recall the day you received the message in the last exam period.

**Please, tell me if you received an e-mail or text message from the University of Szeged before your exam in December 2019?** (only one answer is possible)

1. E-mail
2. SMS
3. I do not remember

*Now, please think about that message that you received first.*

**What was the content of that message?** (only one answer is possible)

1. The message reminded me that I have an exam
2. The message included words of encouragement and wishes for a successful exam
3. The message warned me that I needed to learn
4. The message warned me that I have to return a book to the library.

**How much you believed the content of that message?** (only one answer is possible)

1. I fully believed it
2. I believed it
3. I have partly believed it
4. I have not believed it
5. I have not believed it at all

**How happy were you with the message?** (only one answer is possible)

1. I was very happy
2. I was very happy
3. I was both happy and not happy
4. I was not happy
5. I was not happy at all

**Please, recall when you have not yet received a message from the university. Have you heard of others who have received an encouraging message from the university wishing them good luck with their exams when you have not yet received the encouragement message?** (only one answer is possible)

1. Yes
2. No
3. This has not happened

**How sad you were that, while you did not receive the encouragement message, others received an encouragement message from the university wishing them success in their exams?** (only one answer is possible)

1. It made me very sad
2. It made me sad
3. It made me both sad and not sad
4. It made me not sad
5. It made me not sad at all

**Have you forwarded to others that encouraging message in which the university wished you all success in your exam?** (only one answer is possible)

1. I have not shared the message
2. I have shared the message with my friends at the university
3. I have shared the message with my family

**In the future, would you like to receive similar encouragement messages from the University of Szeged?**

1. Yes
2. No

**References**

1. Sarason IG. Test Anxiety: Theory, Research, and Applications. Hillsdale, NJ: Lawrence Erlbaum Associates; 1980. 193–216 p. Available from: http://www.mrc.stlmath.com/pdf/anxiety/scale.pdf

2. Shrauger JS, Schohn M. Self-Confidence in College Students: Conceptualization, Measurement, and Behavioral Implications. Assessment. 1995 Sep 26;2(3):255–78. Available from: http://journals.sagepub.com/doi/10.1177/1073191195002003006

3. Andrisani PJ. Internal-External Attitudes, Personal Initiative, and the Labor Market Experience of Black and White Men. J Hum Resour. 1977;12(3):308. Available from: https://www.jstor.org/stable/145493?origin=crossref
