# Supplementary material for: Not just words! Effects of a light-touch randomized encouragement intervention on students’ exam grades, self-efficacy, motivation, and test anxiety
Source: PLoS One. 2021 Sep 15;16(9):e0256960. doi: 10.1371/journal.pone.0256960 (PMC8443032; doi:10.1371/journal.pone.0256960)
Supplement: S10 Appendix — (DOCX) [file pone.0256960.s010.docx]

**S10 Appendix: Deviations from the preregistered pre-analysis plan**

The appendix belongs to the following paper by **Tamás Keller** and **Péter Szakál**:

Not just words! Effects of a light-touch randomized encouragement intervention on students’ exam grades, self-efficacy, motivation, and test anxiety

This appendix lists the deviations from the preregistered pre-analysis plan that we archived at the registry for randomized controlled held by the American Economic Association: https://www.socialscienceregistry.org/ on 8th December 2019 before receiving the endline data and before the beginning of the fieldwork.

The preregistration with the detailed analysis plan is available here: <https://doi.org/10.1257/rct.5155-1.1>.

We deviated from our pre-analysis plan in the following respects:

**We changed the term self-concept to self-efficacy**

In our pre-analysis plan, we used the term self-control instead of self-efficacy. In the paper, we use the term self-efficacy, and we refer to the same measured concept. We have changed the term we use but not the underlying empirical concept since our measure refers to the confidence in a specific task (successful exam). As Bandura [1: 382] says: “Confidence is a nondescript term that refers to strength of belief but does not necessarily specify what the certainty is about […] self -efficacy […] includes both an affirmation of a capability level and the strength of that belief.”

**We changed our policy about reporting the results on particular subsamples**

We preregistered to show the results calculated in the subsample of baseline survey data and endline survey data only in the case when these subsamples are a random sample of students. We deviate from this decision in the analysis since (as preregistered) all our estimations controlled for the baseline data, based on which we detected the significant differences between students in different subsamples.

**We changed our policy about reporting the results on those who have only one outcome data**

We preregistered to not analyze students who only had one exam in the semester, and thus could not be treated before their second exam. In the analysis, we deviated from this decision since it would decrease the sample size of the endline survey, which many students answered either before the first or before the second exam. In S7 Appendix, however, we show results for students who have two endline grades (Table A5) and who answered the endline survey twice before both of their exams (Table A6-A8). The results of the tables in the appendix are qualitatively similar to the results shown in the paper.

**We have do not show the results of the preregistered mediation analysis in the main body of the paper**

**The preregistered mediation analysis**

|  | Mediator:  Endline test anxiety | | Mediator:  Endline self-efficacy | | Mediator:  Endline motivation | |
| --- | --- | --- | --- | --- | --- | --- |
|  | (1) | (2) | (3) | (4) | (5) | (6) |
| Treated | 0.069* | 0.068* | 0.053 | 0.021 | 0.061+ | 0.055 |
|  | (0.035) | (0.035) | (0.035) | (0.034) | (0.035) | (0.035) |
| Exam (second =1) | -0.148*** | -0.150*** | -0.148*** | -0.125** | -0.141*** | -0.139*** |
|  | (0.039) | (0.039) | (0.039) | (0.038) | (0.039) | (0.039) |
| Carry-over | -0.084 | -0.083 | -0.073 | -0.073 | -0.084 | -0.078 |
|  | (0.059) | (0.059) | (0.060) | (0.058) | (0.060) | (0.059) |
| Endline test anxiety |  | -0.010* |  |  |  |  |
|  |  | (0.005) |  |  |  |  |
| Endline self-efficacy |  |  |  | 0.108*** |  |  |
|  |  |  |  | (0.006) |  |  |
| Endline motivation |  |  |  |  |  | 0.068*** |
|  |  |  |  |  |  | (0.009) |
| Constant | 3.134*** | 3.208*** | 3.172*** | 2.358*** | 3.317*** | 2.710** |
|  | (0.724) | (0.725) | (0.726) | (0.712) | (0.823) | (0.824) |
| Observations | 8,017 | 8,017 | 7,998 | 7,998 | 8,002 | 8,002 |
| N of students | 6,708 | 6,708 | 6,694 | 6,694 | 6,699 | 6,699 |

All models contain the following preregistered standard baseline control variables: student’s gender, age, ability, student is a first-year student, the type of training, the financial form of training, the level of training, the difficulty of the exam, and study program fixed effects.

Standard errors in parentheses, *** p<0.001, ** p<0.01, * p<0.05, + p<0.1

**References**

1. Bandura A. Self-Efficacy: The Exercise of Control. New York: W. H. Freeman; 1997.
