# Supplementary material for: Not just words! Effects of a light-touch randomized encouragement intervention on students’ exam grades, self-efficacy, motivation, and test anxiety
Source: PLoS One. 2021 Sep 15;16(9):e0256960. doi: 10.1371/journal.pone.0256960 (PMC8443032; doi:10.1371/journal.pone.0256960)
Supplement: Attachment — Submitted filename: Response to Reviewers.docx [file pone.0256960.s012.docx]

Reviewer #1:

This version of the paper is much improved. I enjoyed re-reading it.

I thank the authors for addressing my comments. It is now clearer how they selected the message content, when students received both the email and text messages, and that the length of text messages was fixed. It also now easier for the reader to interpret the parameter, beta 3, as the mean difference in exam grades between treated and control students in the second exam minus this same difference in the first exam. The authors have also done a nice job of toning down the text throughout the paper to better reflect the findings and have nicely explained how they are thinking about the cost-benefit analysis of their intervention.

While these are all great improvements, I do have three remaining concerns about the paper.

***First*, while the mathematical representation of the parameter, beta 3, is now clear, I still do not quite understand how to think about it in the context of a carry-over effect. This parameter is negative in Tables 2 to 4, which means that the difference in outcomes between treated and control students is higher on the first exam than it is on the second exam. Why do the authors think this is the case? What is the hypothesized mechanism?**

**In Table 4, the estimate of beta 3 is negative and, together with beta 1, implies that treatment was ineffective in group B. Is this because the treatment on group A had a persistent effect on exam 2, pushing up the exam 2 scores of group A (relative to what would have been the case without treatment on exam 1)? Or is it because treatment is simply more effective when applied earlier in the semester? It is still unclear to me how to think about the underlying dynamics that result in the estimated value for this parameter. I think the authors could provide more of an explanation than is currently provided between lines 599 and 605 of the manuscript.**

Thank you very much for raising this important point which led us to expand the explanation on the carry-over effect.

The interaction of T and E indicates the carry-over effect, i.e., whether the ordering of the treatment influences the outcome variables. A significant carry-over effect biases the estimation of the average treatment effect.

In our design, we expect a negative carry-over effect, which means that encouraging students before their first exam affects their outcomes at the second exam. Since the sequence of treated and control conditions is either treated-control (Group A) or control-treated (Group B), treating students first might lead to a long-lasting effect or a long wash-out period. A statistically significant negative carry-over effect signals that the treatment effect is higher at students’ first exam than at their second. A negative carry-over effect legitimizes the encouragement treatment and shows that students yearn for encouragement, since treating them before their first exam also affected their outcomes at the second exam, when they were not treated.

Under the current design, the carry-over effect does not provide a substantive interpretation of possible mechanisms that might lead to the longer-lasting effect when treating students before the first exam instead of the second.

***Second*, and related, these issues around beta 3 become especially relevant when it comes to assuaging concerns about selection into the endline questionnaire. The authors say treatment status decreases completion of the endline survey but that Tables A6 to A8 show similar results when the sample is restricted to students who completed the endline survey twice. I respectfully disagree that the results are similar.**

**In particular, Table 3 is one of the most important tables in the paper, highlighting the only effect on one of the secondary outcomes—namely, self-efficacy. (The authors concede that the effect on motivation is less clear because it does not replicate on exam 2.) In Table 3, the treatment effect is large and present on both exam 1 and exam 2. But in Table A7, the analogue to Table 3 but with only students who completed the endline survey twice, treatment appears to influence self-efficacy only on exam 1. That is, beta 3 is negative and significant in nearly all the first seven columns, and the sum of beta 1 and beta 3 is considerably smaller than just beta 1. I cannot tell when the sum remains statistically significant, but it surely does not in all specifications and the resulting treatment effect on exam 2 is always much smaller than the estimate of beta 1 in Table 3. I read the contrast between Tables 3 and A7 as potentially indicating that selection into the endline survey by treatment status is a problem, as Table A7 provides another instance (in addition to Table 4) of the treatment effect not replicating on exam 2.**

We would like to express to you our highest gratitude for the careful reading of the Appendix tables. We realized that we made a severe failure when we copied the results from the output files to the paper. Specifically, we exchanged the exam effect with the carry-over effect. Therefore, your reading of the results based on our wrongly edited paper was correct!

We have cleaned this inconsistency, and the correct appendix tables now show no carry-over effect. Specifically, the results shown in Table 3 and Table A7 are qualitatively similar. The tables can be reproduced and checked since we provided the data and analytical scripts at the OSF platform cited in the paper.

Your comment has inspired us to edit all tables. We included the treatment’s effect concerning the second treatment since it is the linear combination of the coefficients $\beta_{1}$ and $\beta_{3}$. The last row of tables (Table 3-5 and Tables A4-A8) now contains the treatment effect concerning students’ second exam, with the corresponding standard errors.

***Third*, while the authors have done a great job explaining their cost-benefit analysis, I am not convinced that this is a program worth scaling—or at least that this paper provides evidence to that effect.**

**To start, there was no effect on exam grades. This may because there simply was not enough time between the encouragement and the exam for students to change behavior, as the authors point out in the discussion. The authors also note on lines 705 and 706 that grading on a curve may be the reason why exam grades did not go up. It seems that neither explanation warrants an expansion of the program the authors tested. If enough time had not passed between encouragement and the exam, then one should consider an intervention that encourages students earlier or more consistently throughout the semester. But that is not the intervention about which this paper presents evidence. If grading on a curve prevents an effect on exams, then it is unclear how any intervention might work.**

**I agree with the authors that exam grades are not the only (or even the most important) outcome worth considering. The secondary outcomes the authors test are also important. But, as mentioned, I am not convinced that treatment did influence any of the secondary outcomes that the authors explore. Another, related encouragement campaign might, but again, that evidence is not presented here. In sum, I see very little evidence for the benefit side of the cost-benefit analysis for the program studied in this paper.**

Thank you very much for this very insightful argument, which motivated us to delate the policy recommendation concerning scaling up the treatment.

We have reframed our conclusion and write that light-touch, automated encouragement messages, requiring minimal additional human effort from the message provider and sent shortly before exams, do not affect students’ exam grades. Nevertheless, we have isolated a possible mechanism through which encouragement interventions might exert their effect. Specifically, we found that self-efficacy is sensitive to encouraging words, even if students only receive them on an occasional basis shortly before an academically challenging exam situation. Therefore, further encouragement interventions targeting students’ self-efficacy might promote a school climate that boosts students’ engagement in the academic side of school life.

Nevertheless, we made it clear that future encouragement interventions should further improve on our automated encouragement message, which required minimal additional human effort from the message provider. For example, personalized (rather than uniform) messages sent by senders to whom students have contact (e.g., a professor or role model rather than the Head of the Directorate of Education, with whom most students do not have direct contact) could increase the efficacy of future treatments. Furthermore, interventions that encourage students earlier, or more consistently throughout the semester on a systematic rather than occasional basis, should also be considered to increase the treatment effect.

Reviewer #2:

**1. Thank you for your clarifications. I am still confused about how you are describing your models. As I understand it, you had two groups, Group A and Group B. Group A was treated on test 1, Group B was treated on test 2 (except in the small percentage of cases where this did not occur).**

**Your basic model is specified as:**

**Y=beta0+beta1XTreatment+beta2XTest 2+beta3XTreatmentXTest 2+epsilon.**

**As far as I understand it the way you have specified the Treatment variable is 1 for group A for test 1, 0 for group A for test 2, 0 for group B for test 1, 1 for group B for test 2. Is this correct?**

Thank you very much for your consideration, your interpretation is correct.

**The way you describe a carry-over effect it is “a significant carry-over effect reflects that encouraging students before their first exam affects their grades at the second exam.” I think this is one plausible interpretation of beta3 but I do not think it is the only plausible explanation for possible differences. The only accurate way to describe the effects I think is as the difference in the effects between the first and second exam. It could be that the effect differences come from the ordering of the treatments as you say, but another possibility is that there is a difference in the effect due to the difficultly or nature of the first versus the second exam. Perhaps these messages have a different effect later in the semester. Perhaps the messages have more effect on one exam because of the content of one exam versus the other.**

Thank you very much for raising this important point which led us to expand the explanation on the carry-over effect.

The interaction of T and E indicates the carry-over effect, i.e., whether the ordering of the treatment influences the outcome variables. A significant carry-over effect biases the estimation of the average treatment effect.

In our design, we expect a negative carry-over effect, which means that encouraging students before their first exam affects their outcomes at the second exam. Since the sequence of treated and control conditions is either treated-control (Group A) or control-treated (Group B), treating students first might lead to a long-lasting effect or a long wash-out period. A statistically significant negative carry-over effect signals that the treatment effect is higher at students’ first exam than at their second. A negative carry-over effect legitimizes the encouragement treatment and shows that students yearn for encouragement, since treating them before their first exam also affected their outcomes at the second exam, when they were not treated.

Under the current design, the carry-over effect does not provide a substantive interpretation of possible mechanisms that might lead to the longer-lasting effect when treating students before the first exam instead of the second.

We respectfully note that the exam dummy captures all differences concerning students’ first and second exams, including the difference in the first versus second exams’ difficultly or nature.

We clarified that students took their second exam soon after their first exam. The median student had four days between their first and second exams, and most frequently (in 21% of cases), there was only one day between the two exams.

Reviewer #3:

**The authors have responded pretty well to the comments I raised. I am satisfied with the revisions made.**

**I think the manuscript improved due to this revision. I actually do not have any further comments.**

Thank you very much for this positive assessment and evaluation.
