# Supplementary material for: Not just words! Effects of a light-touch randomized encouragement intervention on students’ exam grades, self-efficacy, motivation, and test anxiety
Source: PLoS One. 2021 Sep 15;16(9):e0256960. doi: 10.1371/journal.pone.0256960 (PMC8443032; doi:10.1371/journal.pone.0256960)
Supplement: Attachment — Submitted filename: referee report.pdf [file pone.0256960.s011.pdf]

Referee Report on "Not just words! Effects of encouragement on students' exam grades and non-cognitive skills—lessons from a large-scale randomized field experiment"

A. Paper Summary

This paper experimentally evaluates the effects of sending encouragement messages to students via SMS and email on exam performance, test anxiety, self-confidence, and intention to do well on the exam. The authors conduct the experiment at the University of Szeged with approximately 15,000 students. The encouragement messages are sent to (a randomly selected) half of the students before the first exam and to the other half of the students before the second exam. Students are also asked to complete a three-question survey, asking about anxiety, self-confidence, and intentions, prior to taking both exams. The authors find no effect of receiving the encouragement message on exam performance or test anxiety. They do find that treatment increased students' self-reported self-confidence and intention to do well before the exam.

B. Evaluation

This is a well-motivated, ambitious paper that explores the important question of whether we can improve students' non-cognitive skills cheaply and at scale. The data gathering effort is impressive and the analysis is thoughtful. Indeed, the paper offers many interesting results to contemplate and there is a lot to like about the paper.

I do, however, have several comments and suggestions.

Major Comments:

1. The Messages Students Received.

    a. **General Content Selection**. It would be good to offer more discussion on how the language in the email and text messages was chosen. These messages aim to affect student exam performance by first affecting self-confidence, test anxiety, and intentions to do well. How does the language in the messages affect each of these three traits?

    b. **SMS Content and Length**. Why not include more motivating content into a slightly longer text message and/or include the link to the three-question survey in the text message instead of just the email? My experience is that students are much more likely to engage with text messages than emails.

    c. **Timing of Messages**. Could the authors offer more discussion on when (exactly) students received the messages? Figure 2 is instructive, but I am not sure how to read it. Does it mean that exams could have happened on Dec 9, 2019, Dec 19, 2019, etc.? If so, then there are six exam dates and that would mean that some

students received their message on the day of the exam while others received it up to nine days before the exam or the day after the exam. I would like to know when, relative to the day of the exam, students received the encouragement messages. (Figure 4 implies that the average student completed the three-question survey 13 hours before the first exam. But, again, when were these messages sent to students?) That seems important for thinking about the treatment effect.

2. Estimation and Interpretation of Treatment Effects.

   a. **Main Estimating Equation**. Could the authors spend more time discussing how to interpret the parameters $\beta_1, \beta_2,$ and β_3 in equation 1? I do not believe the interpretation is as easy as the authors make it out to be, given the crossover randomization design where all students are sometimes treated and sometimes control. Here is a basic difference-in-differences style table that one would normally use when time is interacted with treatment status (ignoring other covariates in the model) but considering the crossover design in the current experiment:

	**Group A**	**Group B**	**Difference (A – B)**
**First Exam**	$\beta_0 + \beta_1$	β_0	β_1
**Second Exam**	$\beta_0 + \beta_2$	$\beta_0 + \beta_1 + \beta_2 + \beta_3$	$-\beta_1 - \beta_3$
**Difference (First – Second)**	$\beta_1 - \beta_2$	$-\beta_1 - \beta_2 - \beta_3$	$2\beta_1 + \beta_3$

A couple of challenging questions of interpretation come up:

- β_2: This is the difference between the average **second exam score of group A** and the average **first exam score of group B**. As such, this will only capture the (pure) difference in difficultly between the exams when the persistence of the treatment effect is exactly zero. Otherwise, some of the treatment effect from the first exam will persist into the second exam, affecting the performance of group A on the second exam. At the extreme, suppose treatment perfectly persists to the second exam and the exams are exactly the same level of difficulty. Then we should have $\beta_1 = \beta_2$. Given this, is it not β_2 that captures carry-over effects instead of β_3? Perhaps I misunderstood what is meant by "carry-over".

- β_3: This parameter is difficult to interpret. Isolating it requires summing up the average first and second exam scores for Group B and Group A and then taking the difference between these sums:

$$\beta_3 = (S+F)_B - (S+F)_A$$
$$\beta_3 = (2\beta_0 + \beta_1 + \beta_2 + \beta_3) - (2\beta_0 + \beta_1 + \beta_2)$$

So, then, how do we interpret this parameter?

It may be helpful to report estimates from two separate regressions: one where the first exam is the dependent variable; and another where the second exam is the dependent variable. This would not solve the issues of treatment dynamics and persistence, but it might make it easier for the reader to think about the estimated parameters.

- A general comment on the treatment effects estimation: Ding and Lehrer (2010) is an excellent piece on estimating dynamic treatment effects and interactions between treatment effects in multiple time periods. Doing this fully requires four groups of students: (i) never treated, (ii) treated in period 1 but not 2, (iii) treated in period 2 but not 1, (iv) treated in both periods. Here, the authors do not have a never-treated group or an always-treated group, making it impossible to recover some of the effects I think they would like to recover.

  I may be wrong about how the authors are interpreting the parameters and what is meant by "carry-over" effect. In that case, it would be good for them to clarify.

  Reference:

  Ding, Weili and Steven F. Lehrer. 2010. "Estimating Treatment Effects from Contaminated Multiperiod Education Experiments: The Dynamic Impacts of Class Size Reductions." *The Review of Economics and Statistics*, 92(1): 31-42.

b. **Selection into endline questionnaire**. Table 3 is important and informative, showing no differential selection between groups A and B, except into completing the endline survey twice versus once. But did the authors check whether students were more likely to complete the endline questionnaire before the exam on which they were treated? That is, was group A more likely to complete the endline survey before exam 1 than group B? And was group B more likely to complete the survey before exam 2 than group A?

I do not believe any of the results in Table 3 speak to this (Appendix Tables A7 to A10 do somewhat), although I could be wrong. This type of selection is important to rule out because it would imply students differentially selecting into the survey based on treatment status, calling into question the estimated treatment effects on the items from the survey.

c. **Interpreting Treatment Effects Considering Possible Contamination**. The authors acknowledge the possibility of treatment contamination on pages 5 to 6 and present compelling evidence from the online survey to suggest that contamination likely does not affect the estimates much. But I am wondering why this survey was done five months after the encouragement campaign. Can the authors argue that students are still likely to remember how they felt when they were not receiving messages and their friends were?

d. **Subgroup Effects**. There are many regressions run. Between Tables 4 to 7, there are 48 columns. Table 8 lists 40 hypotheses to be tested. On page 15, the authors acknowledge that the sporadic significant interaction coefficients across these tables could simply arise from multiple testing. I think it would be good to include such cautious language when discussing the interaction coefficients in the body of the paper, too. Otherwise, I think these statements sound too conclusive.

e. **Reported Effects on Student Intentions to Do Well**. Table 7 shows the effect on intentions did not replicate in the second exam because $\beta_1 + \beta_3$ (the difference in average exam 2 scores between group B and group A) is not statistically different from zero. One might read this as a failed replication attempt, depending on how we should think about treatment persistence and time, and so I am not sure why the effect on students' intentions to do well is treated as a headline result (mentioned in the abstract and introduction) when the finding may not replicate within the paper.

3. Cost-Benefit Conclusions.

On page 16, the authors note that their campaign produced cost-effective results on secondary outcomes. Could they provide more evidence for the benefits side of this claim? There are two main concerns. First, as mentioned above, it is questionable whether treatment had any effect on intentions to do well, as this result does not seem to replicate on exam 2. So that only leaves the self-confidence result as robust. Second, then, what is the benefit of the (likely) short-term boost in self-confidence? How does one quantify it and why is it valuable?

<u>Minor Comments</u>:

1. Last sentence of the abstract: “The sporadic treatment effect heterogeneity in the **secondary** outcomes…” The word “secondary” should actually be “primary” I believe.
2. At the end of page 2, the authors say that the study is not specific to a particular sub-population of students. That is true but it is specific to the types of students who attend a given university. I would probably just be more conservative with this statement, as the study does not stretch across many institutions.
3. Page 4, the last sentence of section II.2: in which units is the 0.03 effect size measured when discussing power calculations?
